# Feasibility, usability, and acceptance of “Brain-IT”—A newly developed exergame-based training concept for the secondary prevention of mild neurocognitive disorder: a pilot randomized controlled trial

**DOI:** 10.3389/fnagi.2023.1163388

**Published:** 2023-09-21

**Authors:** Patrick Manser, Hanna Poikonen, Eling D. de Bruin

**Affiliations:** ^1^Motor Control and Learning Group, Institute of Human Movement Sciences and Sport, ETH Zurich, Zurich, Switzerland; ^2^Learning Sciences and Higher Education, Department of Humanities, Social and Political Sciences, ETH Zurich, Zurich, Switzerland; ^3^Department of Health, OST - Eastern Swiss University of Applied Sciences, St. Gallen, Switzerland; ^4^Division of Physiotherapy, Department of Neurobiology, Care Sciences and Society, Karolinska Institute, Stockholm, Sweden

**Keywords:** cognition, cognitive impairment, electroencephalography, exercise, exergaming, neurosciences

## Abstract

**Background:**

Exergames provide a promising new approach to implement simultaneous motor–cognitive training, which may support preventing the decline in cognitive functioning in older adults who have a mild neurocognitive disorder (mNCD).

**Objectives:**

To evaluate feasibility, system usability, and acceptance of “Brain-IT”, a newly developed training concept combining exergame-based motor-cognitive training and heart rate variability (HRV) guided resonance breathing for the secondary prevention of mNCD.

**Methods:**

A pilot randomized controlled trial (RCT) with an allocation ratio of 2:1 (i.e., intervention:control) was conducted. The control group proceeded with usual care. The intervention group performed a 12-week training according to the “Brain-IT” training concept implemented with the “Senso Flex” (Dividat AG) exergaming system in addition to usual care. Feasibility and usability outcomes were analyzed using descriptive statistics. User acceptance was analyzed qualitatively and using Friedman analysis of variance (ANOVA), as well as Wilcoxon signed-rank tests.

**Results:**

Eighteen participants (77.3 ± 9.8 years; 44.4% females) were included. On average, we recruited 2.2 participants per month, and 35.3% of the individuals contacted were included. The intervention group had an attrition rate of 20% and mean adherence and compliance rates of 85.0 and 84.1%, respectively. The mean system usability score, measured with the system usability scale, was 71.7. High levels of exergame enjoyment, an increase in exergame enjoyment, and internalization of training motivation with large effect sizes (*p* = 0.03, r = 0.75 and *p* = 0.03, r = 0.74, respectively), as well as acceptable perceived usefulness, were observed. Preliminary data on the effects of the “Brain-IT” training are promising.

**Conclusion:**

The feasibility and usability of the “Brain-IT” training are acceptable. However, frequent occurrences of technical problems and difficulties in using the exergame training system were identified as barriers to performing the “Brain-IT” training. To optimize feasibility, either improvements or alternative solutions are required in the hardware and software of the exergame used to implement the “Brain-IT” training. The “Brain-IT” training itself was well-accepted by older adults who have mNCD. Therefore, the effectiveness of the “Brain-IT” training concept should be investigated in future studies.

**Trial registration:**

clinicaltrials.gov/ct2/show/NCT04996654.

## 1. Introduction

### 1.1. Background

Preventing disabilities due to cognitive impairment has been declared a public health priority by the World Health Organization (WHO, 2013). Potentially modifiable risk factors for cognitive impairment include diabetes mellitus (Barnes and Yaffe, 2011; Pal et al., 2018; Livingston et al., 2020; Zhang et al., 2022), hypertension (Barnes and Yaffe, 2011; Pal et al., 2018; Livingston et al., 2020; Zhang et al., 2022), obesity (Barnes and Yaffe, 2011; Livingston et al., 2020; Zhang et al., 2022), depression (Barnes and Yaffe, 2011; Livingston et al., 2020; Ma, 2020; Zhang et al., 2022), physical (Barnes and Yaffe, 2011; Livingston et al., 2020; Zhang et al., 2022) or cognitive inactivity (Barnes and Yaffe, 2011), and smoking (Alonso et al., 2009; Barnes and Yaffe, 2011; Livingston et al., 2020; Zhang et al., 2022). Estimates suggest that up to half of the world's cases of Alzheimer's disease (AD)—the leading cause of mild-to-major neurocognitive disorders (m-MNCDs) (American Psychiatric Association, 2013)—may be attributable to modifiable risk factors (Barnes and Yaffe, 2011; Livingston et al., 2020). Lifestyle changes that target these risk factors may hold promise for slowing down cognitive decline or reducing the risk of developing dementia (Kivipelto et al., 2018; Smith, 2019). Physical inactivity is associated with most of the other modifiable risk factors (Barnes and Yaffe, 2011). As an example, physical exercise is effective in reducing cardiovascular risk factors (Liang et al., 2021) and improving depression (Singh et al., 2023) across a very wide range of populations, including mNCD (Leng et al., 2018). Therefore, increasing physical activity may have an impact on m-MNCD prevalence (Barnes and Yaffe, 2011). Additionally, mental stimulation helps build a “cognitive reserve,” which enables individuals to continue functioning at a “normal” level, despite experiencing neurodegenerative changes (Stern, 2002, 2009; Barnes and Yaffe, 2011). In line with the “guided-plasticity facilitation” framework (Fabel and Kempermann, 2008; Kempermann et al., 2010; Herold et al., 2018), combining physical and cognitive training seems the most effective type of training for improving cognitive functioning in older adults who have mNCD (Bruderer-Hofstetter et al., 2018; Biazus-Sehn et al., 2020; Gavelin et al., 2021; Meng et al., 2022). There are different forms of combined motor–cognitive training, including “sequential,” “simultaneous-additional,” and “simultaneous-incorporated” motor–cognitive training (Herold et al., 2018). Incorporating cognitive task(s) into motor task(s) (i.e., “simultaneous-incorporated” motor–cognitive training) seems to be the most promising approach in terms of stabilizing neuroplasticity effects (Herold et al., 2018). This prediction is supported by recent meta-analytic evidence, showing that simultaneous motor–cognitive training was most efficacious for improving cognitive functioning in individuals who have mNCD (Gavelin et al., 2021).

Technological innovations (e.g., exergames) provide new options to engage older adults who have mNCD in simultaneous motor–cognitive training (Dove and Astell, 2017). “*Exergaming is defined as technology-driven physical activities, such as video game play, that requires participants to be physically active or exercise in order to play the game.”* (Witherspoon, 2013). Among the key advantages of exergaming compared to conventional motor–cognitive training is that exergames are highly accepted in individuals who have mNCD and increase or enhance participants' motivation to engage in rehabilitation activities (Zhao et al., 2020). This is of high relevance because motivation (especially intrinsic motivation) has been identified as a key factor for promoting positive behavioral changes (Ryan and Deci, 2000) (e.g., adherence to exercise) in different populations, including healthy adults (Duncan et al., 2010; Teixeira et al., 2012; Wilson et al., 2012; Friederichs et al., 2015; Rhodes and Kates, 2015), healthy older adults (Teixeira et al., 2012; Devereux-Fitzgerald et al., 2016; Behzadnia et al., 2020), and also in individuals with chronic diseases (including cognitive impairment) (Di Lorito et al., 2019; Collado-Mateo et al., 2021). As a result, adherence to exergame-based training is typically high in older adults who have m-MNCD (Zhao et al., 2020; Swinnen et al., 2022). Furthermore, exergaming offers “*the unique opportunity for patients to interact in an enriched environment, providing structured, scalable training opportunities augmented by multi-sensory feedback to enhance skill learning and neuroplasticity through repeated practice”* (Aminov et al., 2018), an additional advantage compared to conventional motor–cognitive training.

Previous systematic reviews and meta-analyses have synthesized consistent positive effects on cognitive functioning favoring exergaming in people who have m-MNCD, although there is considerable variation in exergame-based training (Zhao et al., 2020). However, most previous studies applying exergame-based motor–cognitive training in individuals who have mNCD (earlier called “mild cognitive impairment” (MCI) and incorporated as mNCD into latest Diagnostic and Statistical Manual of Mental Disorders 5th Edition (DSM-5) and the International Classification of Diseases 11th Revision (ICD-XI) (American Psychiatric Association, 2013; Petersen et al., 2014; Sachdev et al., 2014; Sachs-Ericsson and Blazer, 2015; World Health Organization, 2018) have used commercially available exergame systems (Hughes et al., 2014; Delbroek et al., 2017; Anderson-Hanley et al., 2018a,b; Amjad et al., 2019; Jirayucharoensak et al., 2019), which are not specifically designed with purpose beyond play, also referred to as “serious game” (Michael and Chen, 2005; Rego et al., 2010). Valenzuela et al. (2018) argued that commercially available systems may be (too) difficult to use for those with little or no experience with technology because these systems often lack clear instructions, present too much graphical information, and have not been designed and developed to provide optimal training components for the target population and aims of the studies in which they were used (Valenzuela et al., 2018). This points to opportunities for improvement in research and rehabilitation by adapting existing exergames or developing novel exergames and exergame-based training concepts specifically tailored to the requirements and needs of individuals who have mNCD (Manser et al., 2023a). So far, only a few studies have used exergames or exergame-based training concepts that were specifically developed for individuals who have mNCD (Ben-Sadoun et al., 2016; Mrakic-Sposta et al., 2018; Wall et al., 2018; Robert et al., 2021) or older adults who have varied motor and cognitive deficits (including individuals who have mNCD) (Mirelman et al., 2016). These were shown to be safe (no training-related adverse events reported) (Mirelman et al., 2016; Wall et al., 2018), acceptable, and enjoyable (Mrakic-Sposta et al., 2018), while the exergame devices used were shown to have acceptable usability (Ben-Sadoun et al., 2016). These exergames and exergame-based training concepts were developed in collaboration between a research laboratory and a software company (Wall et al., 2018) or based on theoretical considerations (Mirelman et al., 2016) reported in the literature (de Bruin et al., 2010). However, the development process has not been transparently reported (Ben-Sadoun et al., 2016; Mirelman et al., 2016; Mrakic-Sposta et al., 2018; Wall et al., 2018; Robert et al., 2021).

When designing and developing (exergame-based) training concepts, taking the intended users' characteristics, needs, experiences, and perspectives into account seems of crucial importance to ensure the quality and use of the final training concept (Yang et al., 2020; Baquero et al., 2021; Manser and Bruin, 2021). More specifically, a user-centered approach should be adopted (Yang et al., 2020; Baquero et al., 2021), whereas the “*central focus should be the inclusion and active participation of end users from the initial stages of development”* (Baquero et al., 2021). Recently, a theoretical framework was introduced that recommends an interactive and participatory design that explicitly includes end users as well as multidisciplinary teams throughout different iterative cycles of development (Baquero et al., 2021). This theoretical framework, the “*Multidisciplinary Iterative Design of Exergames (MIDE): A Framework for Supporting the Design, Development, and Evaluation of Exergames for Health”* (Li et al., 2020), provides comprehensive, integrative, and specific guidance in the design, development, and evaluation of exergames for older adults on basis of an integrated and multifaceted approach (Manser and Bruin, 2020).

### 1.2. Prior work

On this basis, a novel exergame-based training concept was developed specifically for older adults who have mNCD with the aim to halt and/or reduce cognitive decline and improve quality of life. The training concept was developed on the basis of a structured, iterative, and evidence-based approach based on the MIDE framework (Li et al., 2020). This process allowed the identification of multiple key requirements for exergame design as well as training characteristics that have formed the basis for determining components of the resulting training concept (Manser and Bruin, 2021; Manser et al., 2023a). A detailed description of the rigorous, structured, iterative, and evidence-based design and development process, as well as the resulting “Brain-IT” training concept, was published previously (Manser and Bruin, 2021). Applying such an interactive and participatory design and development process aimed to ensure that the training concept meets the requirements and needs of older adults who have mNCD which fosters feasibility, usability, and acceptance of the approach in “real life” (Manser and Bruin, 2021).

### 1.3. Objectives

The primary objective of this study was to evaluate the feasibility, system usability, and acceptance of the “Brain-IT” project and the “Brain-IT” training concept—a newly developed training concept combining exergame-based motor–cognitive training and HRV-guided resonance breathing for the secondary prevention of mNCD. As a secondary objective, the effects of the “Brain-IT” training on global cognitive functioning, domain-specific cognitive functioning, resting-state cortical activity, spatiotemporal parameters of gait, psychosocial factors, and resting cardiac autonomic regulation were explored.

## 2. Materials and methods

### 2.1. Trial design and study setting

A two-arm, prospective, parallel-group, pilot randomized controlled trial with a 2:1 allocation ratio (i.e., intervention:control) including older adults who have mNCD was conducted between July 2021 and June 2022. The control group proceeded with usual care as provided by (memory) clinics where the participants were recruited. The intervention group performed a 12-week training according to the “Brain-IT” training concept in addition to usual care (see Section Interventions). Unequal randomization was chosen because this pilot trial “*involves new, not established interventions and one of the aims might then be to gain experience in delivering the intervention, in which case it is often better to have as many participants receiving the intervention as is feasible”* (Eldridge et al., 2016). The study was registered at clinicaltrials.gov (NCT04996654) and was reported according to “*The Consolidated Standards of Reporting Trials (CONSORT) 2010 statement: extension to randomized pilot and feasibility trials”* (Eldridge et al., 2016) (Supplementary material 1).

After recruitment and providing written informed consent (see Section Recruitment), participants were screened on eligibility (see Section Eligibility criteria), and pre-measurements were scheduled for all eligible participants. Pre- and post-measurements took place at ETH Hönggerberg (Auguste-Piccard-Hof 1, CH-8093 Zurich) within 2 weeks before starting and after completing the intervention period. All measurements were led by two investigators of our research team trained in the application of the measurement techniques and protocols. Pre- and post-measurements were scheduled to take place at approximately the same time of the day (±2 h) for each participant. To minimize the influence of transient confounding effects on HRV, all participants were additionally instructed verbally and in writing to follow a normal sleep routine the day before the experiment, to avoid intense physical activities and alcohol consumption within 24 h before measurements, and to refrain from coffee, or caffeinated drinks, as well as food consumption at least 2 h before measurements (Laborde et al., 2017). After completing pre-measurements, participants were randomly allocated to the intervention or control group and were instructed about their respective intervention procedures (see Section Interventions). For participants in the intervention group, the exergame device was installed at their homes; they received safety instructions and were familiarized with the exergame training system. Subsequently, the “Brain-IT” training was started (see Section Intervention Group). After completing the 12-week intervention period, post-measurements were performed for both groups.

**Figure 1:**
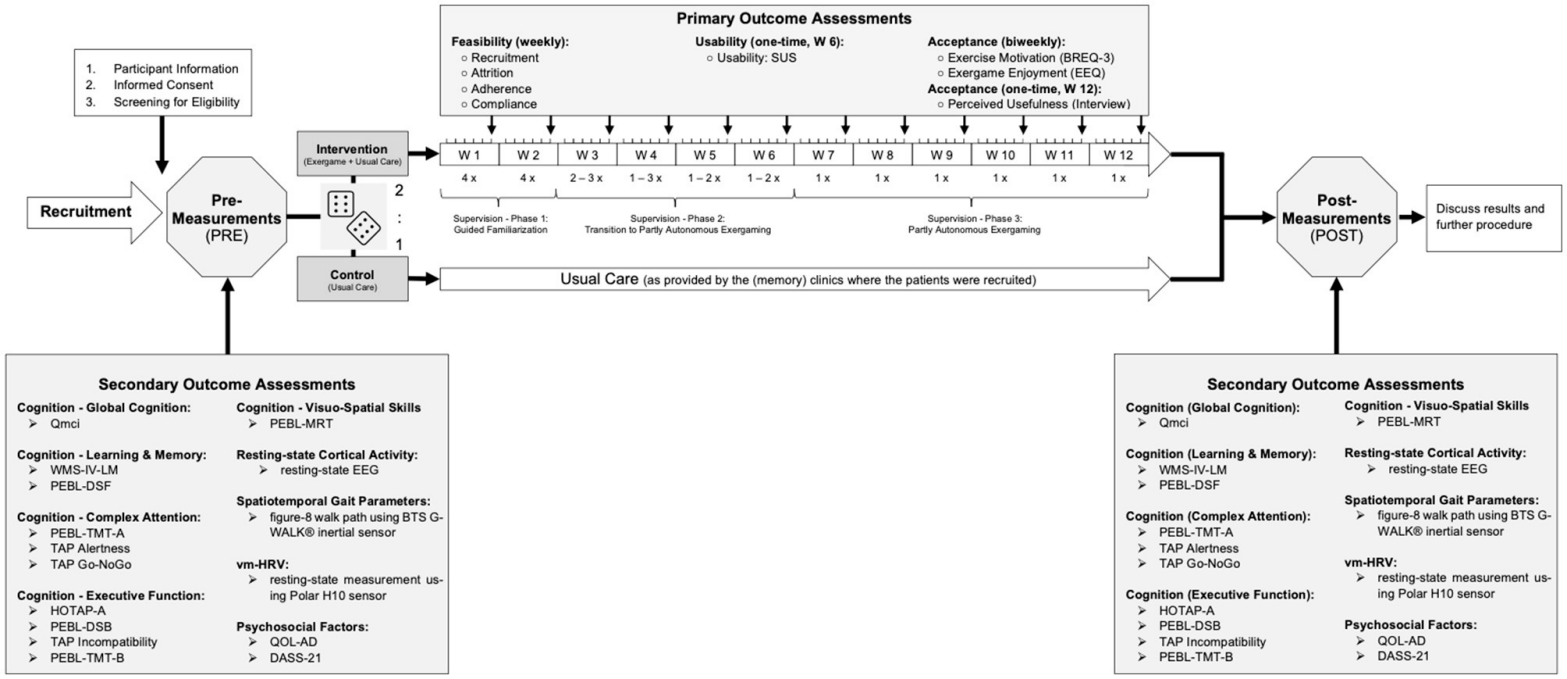
Graphical overview of all study procedures. The cubes are used to illustrate the randomization process [variable block randomization (i.e., block sizes = 3, 6) with a 2:1 allocation ratio (intervention:control) stratified by sex, as described in the Section Randomization]. Qmci, Quick Mild Cognitive Impairment Screen; WMS-IV-LM, Subtest “Logical Memory” of the Wechsler Memory Scale—fourth Edition; PEBL, Psychology Experiment Building Language; DSF, Digit Span Forward; DSB, Digit Span Backward; TMT-A and B, Trail Making Test Part A and B; TAP Alertness, Subtest “Alertness” of the Test of Attentional Performance; TAP Go-NoGo, Subtest “Go-NoGo” of the Test of Attentional Performance; TAP Incompatibility, Subtest “Incompatibility” of the Test of Attentional Performance; HOTAP-A, HOTAP Picture-Sorting Test Part A; MRT, Mental Rotation Task; QOL-AD, Quality of Life-Alzheimer's Disease; DASS-21, Depression, Anxiety and Stress Scale-21; vm-HRV, vagally mediated Heart Rate Variability; SUS, System Usability Scale; BREQ-3, German Version of the Behavioral Regulation in Exercise Questionnaire; EEQ, Exergame Enjoyment Questionnaire.

No compensation was granted to participants, but detailed feedback on individual performance as well as the study outcomes in general was provided at the end of the study. All study procedures were carried out in accordance with the Declaration of Helsinki. The study protocol was approved by the ETH Zurich Ethics Committee (EK 2021-N-79). Figure 1 summarizes the study procedures and outcome measures.

### 2.2. Important changes to the trial design and study setting after commencement

The study was planned as single-blinded (i.e., outcome evaluator of pre- and post-measurements blinded to group allocation) pilot RCT. Due to COVID-19-related delays in recruiting participants, the study period had to be extended. This resulted in personnel changes in the team of study investigators. Consequently, blind keeping of outcome assessors was only possible for approximately half of post-measurements.

### 2.3. Recruitment

Older adults who have mNCD were recruited between July 2021 and June 2022 in collaboration with (memory) clinics in the larger area of Zurich. Suitable individuals were either identified from medical records and patient registries of (memory) clinics or from recent diagnostics performed by their medical doctors or therapists authorized to search medical records. Alternatively, suitable individuals were identified by an informant (i.e., healthcare professionals)-based suspicion of MCI (see Section Eligibility Criteria). Identified individuals were verbally informed about the existence of the study and received leaflets from their physicians/therapists containing key information about study participation and the researchers' contact details. In case the individuals were interested in being informed about the study in detail, they were asked to provide consent to share their contact details with the research team and were contacted by phone or e-mail by a trained investigator of the study team. In case of initial interest in participating in the study, all interested subjects were fully informed about the study procedures in-person [at the interested persons' home or at the study center (ETH Hönggerberg), depending on their preferences] by providing verbal explanations and an information sheet. After sufficient time for consideration (i.e., at least 24 h after handing out the study information sheet, but on average around 1 week), suitable individuals willing to take part in the study provided written informed consent in a second in-person meeting. Subsequently, participants were fully screened on eligibility (see Section Eligibility criteria), and pre-measurements were scheduled.

### 2.4. Eligibility criteria

All eligibility criteria are detailed in Table 1.

**Table 1:** Description of all eligibility criteria.

**Inclusion criteria**	**Exclusion criteria**
**Participants fulfilling all the following inclusion criteria were eligible**	**The presence of any of the following criteria led to exclusion**
• (1 = mNCD) clinical diagnosis of “mild neurocognitive disorder” according to the International Classification of Diseases 11th Revision (ICD-XI) (World Health Organization, 2018) or the latest Diagnostic and Statistical Manual of Mental Disorders 5th Edition (DSM-5^®^) (American Psychiatric Association, 2013) OR (2 = sMCI) individuals screened for mild cognitive impairment (sMCI) according to the following criteria: (a) informant (i.e., healthcare professionals)-based suspicion of mild cognitive impairment (MCI) confirmed by (b) an objective screening of MCI based on the German Version of the Quick Mild Cognitive Impairment Screen (Qmci) (O'Caoimh, 2015) with (b1) a recommended cutoff score for cognitive impairment (MCI or dementia) of < 62/100 (O'Caoimh et al., 2017), while (b2) not falling below the cutoff score for dementia (i.e., < 45/100; O'Caoimh et al., 2017), while (c) activities of daily living remain intact (judged by the referring healthcare professionals). • Fully vaccinated against coronavirus (SARS-CoV-2) with a Swiss Federal Office of Public Health (FOPH)-approved vaccine (BAG, 2021). • German speaking. • age ≥ 50 years. • able to stand for at least 10 min without assistance.	• Mobility impairments (i.e., gait and balance) that prevent experiment participation • Presence of additional, clinically relevant (i.e., acute and/or symptomatic) neurological disorders (i.e., epilepsy, stroke, multiple sclerosis, Parkinson's disease, brain tumors, or traumatic disorders of the nervous system) • Presence of any other unstable or uncontrolled diseases (e.g., uncontrolled high blood pressure, progressing or terminal cancer) Additional *COVID*-19-specific exclusion criteria: Coronavirus Disease 2019 (COVID-19) specific risk factors (according to the Swiss FOPH) were additional exclusion criteria. In the case of COVID-19-specific exclusion criteria, participation in the study was only allowed when the participants' treating physician provided written informed consent allowing participation in the study despite the presence of COVID-19-specific exclusion criteria. COVID-19-specific exclusion criteria included: • High blood pressure (self-reported; systolic ≥140 mmHg and/or Diastolic ≥90 mmHg). • Chronic respiratory condition. • Uncontrolled type 2 Diabetes. • Condition or therapy that weakens the immune system. • Unstable cardiovascular disease. • Cancer (present and/or under treatment). • Serious obesity (body mass index ≥40 kg/m^2^).

### 2.5. Interventions

#### 2.5.1. Control group

The control group proceeded with usual care as provided by the (memory) clinics where participants were recruited. Usual care of mNCD typically includes treating medical conditions other than mNCD (e.g., diabetes mellitus and depressive symptoms), controlling comorbidities (e.g., hypertension and obesity), and managing risk factors (e.g., smoking habits and physical and cognitive inactivity). With this regard, usual care may include medication, recommendations for changing lifestyle habits (e.g., living a cognitively, physically, and socially active life), physiotherapy to treat specific health problems such as back pain or mobility problems, occupational therapy, or day clinic visits. Usual care is highly individual, which varies between (memory) clinics where participants are recruited, and it is unclear whether participants comply with the recommendations of their clinicians. Therefore, details about all structured and/or guided usual care activities as well as medication intake were assessed in both the intervention and the control groups.

#### 2.5.2. Intervention group

**Figure 2:**
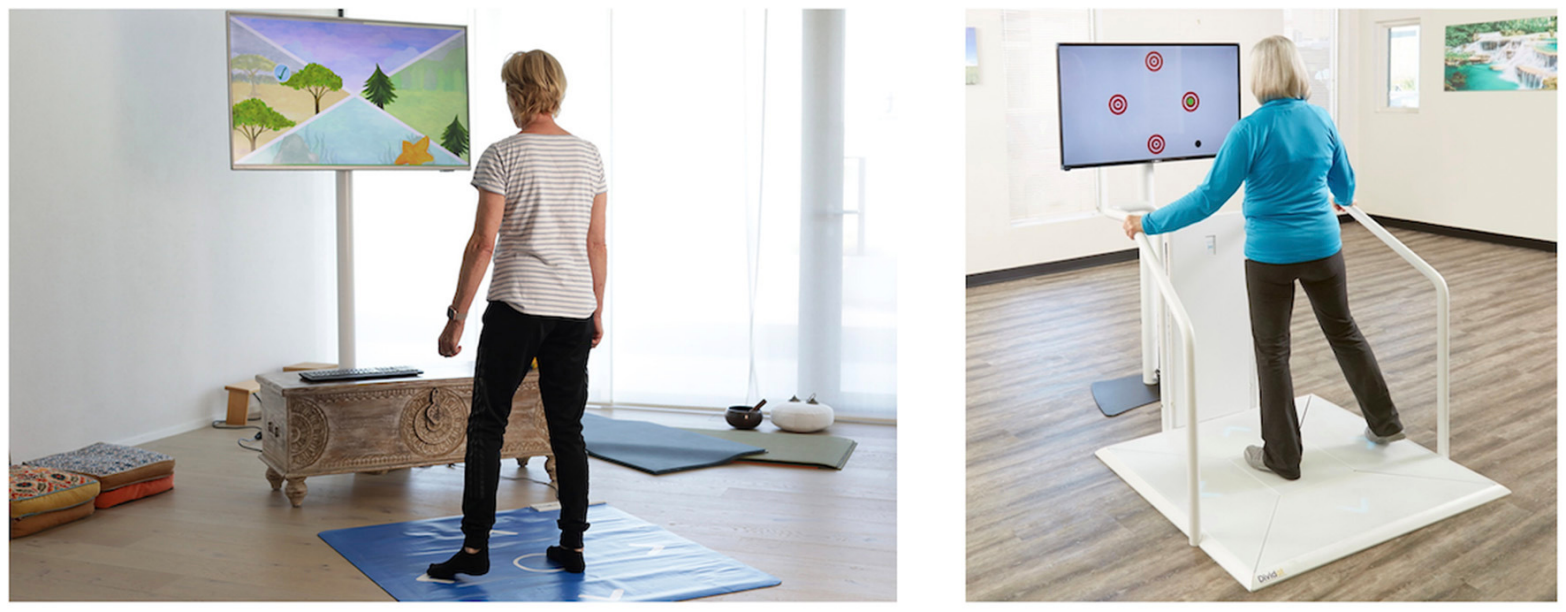
Exergame Device used as means to implement the “Brain-IT” training concept in this study: “Senso Flex” for home-based use **(left side)** and its stationary version [“Senso” for stationary use in physiotherapies, nursing homes, or rehabilitation clinics **(right side)**]. Photos provided by Dividat AG.

Participants in the intervention group performed a 12-week training in addition to their usual care (as provided by the (memory) clinics where participants are recruited). The training was prescribed according to our “Brain-IT” training concept. This training concept represents a guideline for applying a combination of exergame-based motor–cognitive training and HRV-guided resonance breathing by standardizing the training characteristics (e.g., training frequency, intensity, and duration), as well as the structure and content of training, whereas the exergame device and the specific games used within each of the defined neurocognitive domains can be replaced by alternative exergames. Our training concept is implemented using the “Senso (Flex)” (Dividat AG, Schindellegi, Switzerland, CE certification pending; see Figure 2 **left side**). This platform was found suitable to implement our training concept (Manser et al., 2023a) and is a widely used means for motor–cognitive training within geriatric populations, physiotherapies, or rehabilitation in Switzerland. The original “Brain-IT” training concept has recently been published with sufficient detail to allow full replication (i.e., consider Supplementary file 3 of Manser and Bruin, 2021). To ensure replicability, the “Brain-IT” training concept was planned and reported using the Consensus on Exercise Reporting Template (CERT) (Slade et al., 2016).

For an overview, the “Brain-IT” training concept consists of an individually adapted multi-domain exergame-based simultaneous motor–cognitive training with incorporated cognitive tasks combined with HRV-guided resonance breathing. It is adopted with a deficit-oriented focus on the neurocognitive domains of (1) learning and memory, (2) executive function, (3) complex attention, and (4) visuospatial skills. Each participant was instructed to train ≥5x/week for ≥21 min per session resulting in a weekly exercise volume of ≥105 min. All training sessions were planned to take place at participants' homes using the “Senso Flex” hardware. The “Senso Flex” is a home-based version of the “Senso” (Dividat AG, Schindellegi, Switzerland; CE certification; see the right side of Figure 2). It consists of a 1.11 m x 0.99-m rollable mat that is plugged into the portable computer and a frontal television (or other screen) at home. Both systems divide the pressure-sensitive stepping area into five fields: (1) center (home position), (2) front, (3) right, (4) back, and (5) left. The device detects participants' position and timing of movements to interact with different game scenarios that are programmed in the Dividat training software. Weight shifting, walking on the spot, and steps in four directions (i.e., front, right, back, and left) enable interaction with and control of virtual exergame scenarios that are displayed on a screen right in front of the participant. Visual, auditory, and somatosensory (vibrating platform; only available on the “Senso”) feedback is provided in real-time to enrich the game experience. Various games are available to train different neurocognitive domains (for more detail on how the device is implemented in our training concept, see Manser and Bruin, 2021).

As per the “Brain-IT” training concept (Manser and Bruin, 2021), 19–24 training sessions were supervised by a designated investigator who instructed and oversaw the participants' use of the exergame device, ensured safety protocols were followed [e.g., ensuring that there were no hard objects (e.g., couch table) within the potential drop zone, determining the appropriate level of stability support using walking sticks, handrail or similar], and ensured adherence to the “Brain-IT” training concept. All deviations from the “Brain-IT” training concept were reported.

### 2.6. Outcomes

#### 2.6.1. Primary outcomes

##### 2.6.1.1. Feasibility

**Figure 3:**
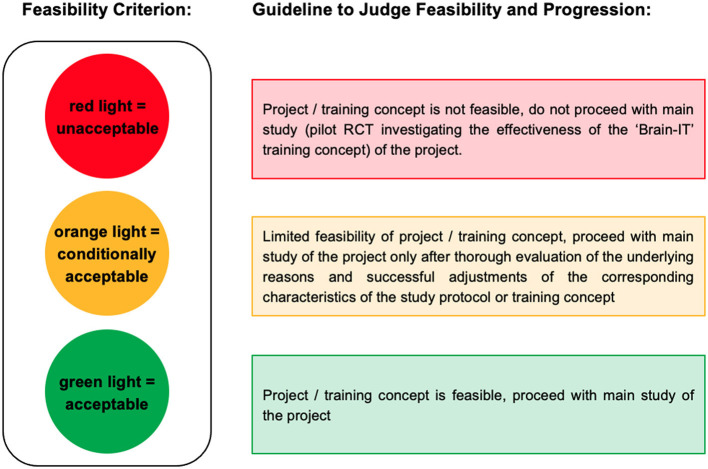
Traffic light system as a guideline to judge feasibility and progression.

The feasibility of the “Brain-IT” project and the “Brain-IT” training was assessed with respect to recruitment, adherence, compliance, and attrition. These endpoints were recorded by a recruitment protocol, automatically assessed in the exergame training software (i.e., adherence and compliance protocol), and detailed electronic case report forms (CRFs) throughout the study period. Feasibility outcomes and their calculation are defined in Table 2. Adherence is usually calculated as “*the proportion between the number of sessions attended and the number of sessions offered, reported in percentage”* (Di Lorito et al., 2020). To ensure that participants who trained more than the prescribed minimum frequency did not compensate for lower adherence and compliance rates in other participants or training weeks in which they trained less, mean adherence and compliance rates were calculated as the average of each participant's weekly adherence/compliance with a maximum of 100%. Reasons for non-adherence, non-compliance, and dropouts were recorded. A traffic light system with quantitative thresholds was used as a guideline to judge feasibility and progression (Figure 3).

**Table 2:** Traffic light system with quantitative thresholds as guideline to judge feasibility and progression.

**Feasibility outcome**	**Calculation**	**Feasibility criteria**		
		**Green light** = **acceptable**	**Orange light** = **conditionally acceptable**	**Red light** = **unacceptable**
Recruitment (absolute)	Absolute recruitment rate (REC_ab_) [] = number of included and eligible participants recruited per month	REC_ab_ ≥ 6/month	6/month ≤ REC_ab_ ≥ 2/month	REC_ab_ ≤ 2/month
Recruitment (relative)	Relative recruitment rate (REC_rel_) [%] = number of contacted individuals/number of included and eligible participants	REC_rel_ ≥ 25%	25% ≤ REC_rel_ ≥ 5%	REC_rel_ ≤ 5%
Adherence	Adherence rate (ADH) [%] = number of training sessions attended/total number of training sessions offered; calculated as the average of each participant's weekly adherence with a maximum of 100%	ADH ≥ 75%	75% ≤ ADH ≥ 50%	ADH ≤ 50%
Compliance	compliance rate (COMP) [%] = training duration attended [min]/total training duration offered [min]); calculated as the average of each participant's weekly adherence with a maximum of 100%	COMP ≥ 75%	75% ≤ COMP ≥ 50%	COMP ≤ 50%
Attrition	Attrition rate (ATT) = number of dropouts/number of included participants who were randomly allocated to the intervention or control group and started the intervention period	ATT ≤ 20%	20% ≤ ATT ≥ 40%	ATT ≥ 40%

REC_ab_, absolute recruitment rate; REC_rel_, relative recruitment rate; ADH, adherence rate; COMP, compliance rate; ATT, attrition rate.

Quantitative thresholds for each feasibility criterion were determined based on an educated guess before starting recruitment as follows: To reach a green light (=acceptable), the mean value of the feasibility outcome (F_mean_) needed to exceed (for attrition rate: fall below) the pooled average of comparable [i.e., exergame or alternatively (combined) physical and/or cognitive exercise) intervention studies based on a recent (within the last 10 years) systematic synthesis of evidence in older adults who have m-MNCD (defined as first threshold (T1)]. The variance of the pooled average was used to determine the lower acceptable threshold [defined as the second threshold (T2)]. In case the mean value of the feasibility outcome fell below (for attrition rate: exceeded) T2, a red light (=unacceptable) was assigned. For values ranging between T1 and T2, an orange light (=conditionally acceptable) was assigned.

The ability to recruit sufficient eligible participants within an appropriate timeframe is crucial for the feasibility of a future RCT. An absolute recruitment rate of at least six eligible participants per month was considered optimal, while two eligible participants per month were considered a minimal requirement for the “Brain-IT” project. Regarding the relative recruitment rate, a median of 26% (range: 3.4–59%) was determined for exergame-based training in individuals who have mNCD in the studies analyzed in Zhao et al. (2020). Based on this information, T1 was set to 6/month for the absolute recruitment rate and 25% for the relative recruitment rate. T2 was set to 2/month for the absolute recruitment rate and 5% for the relative recruitment rate. Adherence and compliance to exergame-based training are typically high in healthy older adults (HOA) (Valenzuela et al., 2018) and older adults who have m-MNCD (Zhao et al., 2020; Swinnen et al., 2022). For individuals who have m-MNCD, mean adherence rates of 70% (standard deviatio*n* = 21, range = 16–100%) (Di Lorito et al., 2020) up to 90% (10th percentile = 79%; 90th percentile = 99%) (Panza et al., 2018) and a median compliance rate of 75% (range: 16–100%) (Di Lorito et al., 2020) to physical training were synthesized. For exergame-based training, a mean adherence rate of 84% (range 69–100%) was reported (Swinnen et al., 2022) and a median compliance rate of 70% (range: 56–100%) was determined for the studies analyzed in Zhao et al. (2020). Based on this information and considering the high training frequency prescribed in this study, T1 was set to 75% and T2 to 50% for both adherence and compliance. Regarding attrition, a mean attrition rate of 17% (range 0–59%) (Di Lorito et al., 2020) was synthesized for physical training and 15% (range: 0–31%) (Swinnen et al., 2022) for exergame-based training in individuals who have m-MNCD. Based on this information, T1 was set to 20% and T2 to 40%.

The resulting traffic light system with quantitative thresholds as a guideline to judge feasibility and progression is illustrated in Table 2.

##### 2.6.1.2. Usability

Usability was assessed by self-report using the validated German version of the System Usability Scale (SUS) (Brooke, 1995; Gao et al., 2020), which is a valid and reliable scale for evaluating newly developed devices and systems (Brooke, 1996; Gao et al., 2020; Albert and Tullis, 2022). It is a frequently used scale for the evaluation of software products and also (exer)games and provides a global view of subjective assessments of usability (Albert and Tullis, 2013). A total score was calculated according to the scoring guidelines of the SUS. Total SUS scores range between 0 and 100, whereas higher scores indicate better usability (Brooke, 1995). A total SUS score of ≥70 was defined as a criterion for “acceptable” usability (Bangor et al., 2009).

##### 2.6.1.3. Acceptance

User acceptance of the newly developed exergame-based training concept was assessed with respect to exergame enjoyment, training motivation, and perceived usefulness.

Exergame enjoyment was assessed biweekly by self-report using the Exergame Enjoyment Questionnaire (EEQ) (Fitzgerald et al., 2020). The German version of the EEQ was used, which has shown good internal consistency and is responsive to changes in differing conditions of exergame enjoyment (Manser et al., 2023b). A total score was calculated according to the scoring guidelines, resulting in a minimum score of 20 and a maximum score of 100. A higher score reflects greater enjoyment of playing the exergame (Manser et al., 2023b).

Training motivation was assessed by self-report using the German translation (Rausch Osthoff, 2017) of the revised (Markland and Tobin, 2004) Behavioral Regulation in Exercise Questionnaire (BREQ) (Mullan et al., 1997), a widely used, valid, and reliable measure of training motivation (Mullan and Markland, 1997; Mullan et al., 1997; Wilson et al., 2002; Markland and Tobin, 2004; Teixeira et al., 2012) along the Self-Determination Continuum (Mullan et al., 1997; Ryan and Deci, 2002). As an outcome measure, the self-determination index (SDI) was calculated as described in Vallerand and Toward (1997). The SDI ranges between −24 and +24, whereas higher positive values represent a higher degree of self-determined motivation (Vallerand and Toward, 1997).

Perceived usefulness was evaluated after the last supervised training session based on individual interviews, organized as semi-structured in-depth interviews (Dicicco-Bloom and Crabtree, 2006) along with an interview guide (Supplementary material 2). In addition to perceived usefulness, the interview guide also contained questions about participants' experiences with the training and desired adaptations of the training concept and/or the exergame device. With this, we aimed to collect data for justifying specific modifications of the “Brain-IT” training concept and/or the exergame device based on the participants' perspectives. Data collection and analysis were done similarly to the methods described in a previous qualitative study within the “Brain-IT” project (Manser et al., 2023a), which included qualitative content analysis according to Mayring (2014) and Mayring and Fenzl (2019) performed using QCAmap software (Mayring, 2014, 2020; Fenzl and Mayring, 2017).

#### 2.6.2. Secondary outcomes

As secondary outcomes, changes in global cognitive functioning and key neurocognitive domains [as defined in Sachdev et al. (2014) in line with DSM-5 (American Psychiatric Association, 2013) and according to recommendations (Janelidze et al., 2018)] of (1) learning and memory, (2) complex attention, (3) executive function, and (4) visuospatial skills, as well as resting-state cortical activity, spatiotemporal parameters of gait, psychosocial factors [i.e., quality of life (QoL), and levels of depression, anxiety, stress], and cardiac vagal modulation [resting vagally mediated HRV (vm-HRV)] were assessed. An overview of all secondary outcome measures is provided in Table 3. Details on specific assessments and measurement conditions of all secondary outcomes are provided in Supplementary material 3.

**Table 3:** Overview of all secondary outcome measures, outcome variables, and interpretation guide.

**Outcome measures**		**Outcome variables**	**Interpretation guide**
Primary:	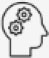 Global cognition		
	Quick mild cognitive impairment screen (O'Caoimh, 2015; O'Caoimh and Molloy, 2017)	Total point score []	Improvement = ↑
Secondary	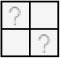 Learning and memory		
	Subtest “logical memory” of the Wechsler Memory Scale—fourth edition (Wechsler, 2009; Petermann and Lepach, 2012)	Total point score part 1—free recall []	Improvement = ↑
		Total point score part 2—free recall []	Improvement = ↑
		Total point score part 2—recognition []	Improvement = ↑
	PEBL Digit Span Forward (Croschere et al., 2012; Mueller, 2014; Mueller and Piper, 2014)	Total point score []	Improvement = ↑
		Maximum span []	Improvement = ↑
	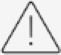 Complex Attention		
	PEBL trail making test—part A (Mueller and Piper, 2014)	Completion time [s]	Improvement = ↓
		Number of errors []	Improvement = ↓
	Subtest “alertness” of the test of attentional performance (Zimmermann and Fimm, 2012)	Median reaction time for condition A [ms]	Improvement = ↓
		Median reaction time for condition B [ms]	Improvement = ↓
	Subtest “Go-NoGo” of the test of attentional performance (Zimmermann and Fimm, 2012)	Median reaction time [ms]	Improvement = ↓
		Number of errors []	Improvement = ↓
	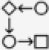 Executive function		
	HOTAP picture-sorting test part A (Menzel-Begemann, 2009)	COMBI score (i.e., sum of the points divided by the time they needed to arrange the cards) [points · min^−1^]	Improvement = ↑
	PEBL digit span backward (Croschere et al., 2012; Mueller, 2014; Mueller and Piper, 2014)	Total point score []	Improvement = ↑
		Maximum span []	Improvement = ↑
	Subtest “incompatibility” of the test of attentional performance (Zimmermann and Fimm, 2012)	Median reaction time condition “compatible” [ms]	Improvement = ↓
		Median reaction time condition “incompatible” [ms]	Improvement = ↓
		Number of errors [] condition “compatible” [ms]	Improvement = ↓
		Number of errors [] condition “incompatible” [ms]	Improvement = ↓
	PEBL trail making test—part B (Mueller, 2014; Mueller and Piper, 2014)	Completion time [s]	Improvement = ↑
		Number of errors []	Improvement = ↑
	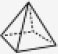 Visuospatial skills		
	PEBL mental rotation task (Berteau-Pavy et al., 2011; Mueller, 2014; Mueller and Piper, 2014)	Median reaction time of correct answered trials [ms]	Improvement = ↓
		Performance (number of correct answered trials) []	Improvement = ↑
	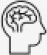 Resting-state cortical activity		
	Resting awake state measurement (two repeats of 2 min eyes closed, 2 min eyes opened, resulting in a total measurement duration of 8 min) using a high-density 64-channel electroencephalography system (eego sport, ANT Neuro, Enschede, The Netherlands). The electrode placement scheme by ANT Neuro (an extension to the 10/20 and 10/10 systems) was used (Chatrian et al., 2015).	Mean beta (13–30 Hz) frequency band amplitude power of Cz [uV^2^/Hz]	Improvement = ↓
		Mean theta (4–8 Hz) frequency band amplitude power of T7 [uV^2^/Hz]	Improvement = ↓
		Mean theta (4–8 Hz) frequency band amplitude power of T8 [uV^2^/Hz]	Improvement = ↓
		Mean theta (4–8 Hz) frequency band amplitude power of FT7 [uV^2^/Hz]	Improvement = ↓
		Mean theta (4–8 Hz) frequency band amplitude power of FT8 [uV^2^/Hz]	Improvement = ↓
		Phase synchrony index of alpha (8–13 Hz) frequency between Fp2-C4 []	Improvement = ↑
		Phase synchrony index of alpha (8–13 Hz) frequency between F7-T6 []	Improvement = ↑
		Phase synchrony index of alpha (8–13 Hz) frequency between T3-T6 []	Improvement = ↑
		Phase synchrony index of alpha (8–13 Hz) frequency between T5-T6 []	Improvement = ↑
	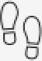 Spatiotemporal parameters of gait		
	Instrumented gait analysis using a figure of eight walking path (Bioengineering, 2017) at preferred walking speed using BTS G-WALK^®^ (BTS Bioengineering S.p.A., Garbagnate Milanese, Italy) inertial sensor attached with the semi-elastic belt to the lower back of the participant.	Walking speed [m · s^−1^]	Improvement = ↑
		Stride duration [ms]	Improvement = ↓
		Stride length [cm]	Improvement = ↑
		Stance phase duration [% stride duration]	Improvement = ↓
		Swing phase duration [% stride duration]	Improvement = ↑
		Single support time [%]	Improvement = ↑
		Double support time [%]	Improvement = ↓
	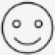 Psychosocial factors		
	Quality of life-Alzheimer's disease (Gibbons et al., 1999; Trust, 2016; Stypa et al., 2020)	Overall score []	Improvement = ↑
	Depression, anxiety, and stress scale-21 (Lovibond and Lovibond, 1995, 1996; Henry and Crawford, 2005; Nilges and Essau, 2015, 2021)	Overall score—subscale depression []	Improvement = ↓
		Overall score—subscale anxiety []	Improvement = ↓
		Overall score—subscale stress []	Improvement = ↓
	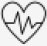 Resting vagally mediated heart rate variability		
	5 min resting vm-HRV measurement with heart rate monitor (Polar M430) and sensor (Polar H10) analyzed using Kubios HRV Premium (Kubios Oy, Kuopio, Finland, version 3.4) (Tarvainen et al., 2014)	Mean R-R time interval [ms]	Improvement = ↑
		Root mean square of successive RR interval differences []	Improvement = ↑
		Percentage of successive RR intervals that differ by more than 50 ms [%]	Improvement = ↑
		Absolute power of the high-frequency (0.15–0.4 Hz) band [ms^2^]	Improvement = ↑
		Relative power of the high-frequency (0.15–0.4 Hz) band [nu]	Improvement = ↑
		Poincaré plot standard deviation perpendicular to the line of identity [ms]	Improvement = ↑
		Parasympathetic nervous system tone index []	Improvement = ↑

↑ = higher values/an increase over time indicate better functioning/improvement in the respective study endpoint. ↓ = lower values/a decrease over time indicate better functioning/improvement in the respective study endpoint. PEBL, Psychology experiment building language; vm-HRV, vagally mediated heart rate variability.

#### 2.6.3. Other endpoints

##### 2.6.3.1. Safety endpoint variables

A protocol was kept for all (serious) adverse events [(S)AEs].

##### 2.6.3.2. Baseline factors

Baseline factors were collected through demographic data including age, sex, height, weight, body mass index (BMI), years of education, physical activity behavior (i.e., time spent in at least a moderate level of physical activity per week), medication intake (yes/no), and etiological subtype (i.e., mainly mNCD due to AD, mild frontotemporal NCD, mNCD with Lewy Bodies, or mild vascular NCD).

### 2.7. Sample size

The sample size was justified based on the rules of thumb of Julious (2005), who recommended a minimum sample size of 12 per group for pilot or feasibility studies (Julious, 2005). As described in the Section Trial design and study setting, the focus of this study was on investigating the primary outcomes in the group receiving our new “Brain-IT” training. Considering the 2:1 allocation ratio, we targeted a sample size of 12 for the intervention and six for the control group, leading to a total sample size of *n* = 18. To ensure an adequate number of participants in the study, a safety margin for an attrition rate of up to 40% (criterion for orange light; see the Section Feasibility) was chosen. Based on these considerations, we aimed to recruit a total of 18–25 participants.

### 2.8. Randomization

#### 2.8.1. Sequence generation

Participants were randomly allocated to the intervention or control group. A variable block randomization (i.e., block sizes = 3, 6) with a 2:1 allocation ratio (intervention:control) stratified by sex was used.

#### 2.8.2. Allocation concealment mechanism

To ensure allocation concealment, the random allocation was computer-generated using a validated variable block randomization model implemented in the data management system Castor EDC (Ciwit BV, Amsterdam, The Netherlands) (Castor, 2019).

#### 2.8.3. Implementation

The randomization process was set up by PM before starting the recruitment of participants. PM was also in charge of the enrollment of participants. Participants were randomly assigned to the intervention or control group by the investigator assigned as the responsible person for supervision and correspondence with the respective participant after completing pre-measurements.

### 2.9. Data management

All involved study investigators were thoroughly trained for all study procedures according to the Guidelines of Good Clinical Practice (GCP) and in line with detailed working instructions. The principal investigator was in charge of methodological standards and quality of data collection using the data management system Castor EDC (Ciwit BV, Amsterdam, The Netherlands). Range checks for data values were pre-programmed for data entry in eCRFs. All data entries were cross-checked by a second study investigator before export for analysis. To minimize bias during the assessment of all outcome measures, detailed working instructions were prepared that included standardized measurement procedures and standardized instructions for participants for all measurements.

### 2.10. Blinding

As clarified in the Section Important changes to the trial design and study setting after commencement, the study was planned as a single-blinded pilot RCT. However, we were not able to keep all outcomes assessors blinded due to COVID-19-related delays in recruiting participants. For all data assessed throughout the intervention period (i.e., only applicable for the intervention group), blinding of investigators was not possible. Blinding of participants was also not possible since usual care was used as a control intervention.

### 2.11. Participant retention

Once a participant was included, a trained investigator was assigned as the person responsible for supervision and correspondence with the respective study participant and made all reasonable efforts to achieve the participant's retention in the study. Examples include providing written information sheets and reminders about study appointments, involving carers or relatives as personal support for study participants, and providing assistance with travel to the study center. Specifically, in the intervention group, each participant was provided with a detailed training manual that was individually adapted to the participants' setup to help them use the training system correctly [with photographs and explanations for each step from starting the system to training completion, including a colored step-by-step identification of required elements (cables and buttons)]. Furthermore, the study team provided telephone support in case of technical difficulties or comprehension problems for unsupervised training sessions.

### 2.12. Statistical methods

Statistical analysis was executed using R Version R 3.6.2 GUI 1.70 El Capitan build (7735) (© The R Foundation) in line with RStudio Version 2022.07.1 (RStudio, Inc.). For demographics and primary outcomes (except user acceptance), all collected data were included (i.e., including data on dropouts up to the timepoint of their withdrawal). For user acceptance, only data of participants who completed the study were analyzed. For all secondary outcomes, a modified intention-to-treat analysis was performed (i.e., data from all participants who completed pre- and post-measurements, regardless of protocol adherence, were included in the data analysis). Questionnaire scores were regarded as ordinal data. Data were reported as mean ± standard deviation for parametric data, median (interquartile range) for non-parametric data, and the frequency of various statements (f) and the proportion of participants making a statement (in %) for qualitative data.

For all outcomes, descriptive statistics were computed first. The normal distribution of data was checked using the Shapiro–Wilk test. The level of significance was set to *p* ≤ 0.05 (two-sided, uncorrected).

For all demographic variables, between-group differences (i.e., intervention vs. control) were tested using an independent *t-*test or Mann–Whitney *U*-test in case the data were not normally distributed. Between-group differences in categorical variables were tested using Fisher's exact test. Feasibility and usability outcomes were analyzed using descriptive statistics and according to predefined criteria (see Section Primary outcomes). User acceptance was analyzed qualitatively (i.e., perceived usefulness) and based on a Friedman ANOVA to evaluate the effect of time on exergame enjoyment and training motivation. Additionally, a Wilcoxon signed-rank test was performed to evaluate whether there was a difference in median exergame enjoyment and training motivation between the first and the last measurement. To discover whether effects were substantive, effect sizes r were calculated (Rosenthal, 1991; Field et al., 2012) and interpreted to be small (0.1 ≤ r < 0.3), medium (0.3 ≤ r < 0.5), or large (r > 0.5) (Cohen, 1988).

For all secondary outcomes, the assumption of homogeneity of variance was checked using Levene's test. In case all assumptions for ANCOVA were met, effects of the addition of the “Brain-IT” training to usual care as compared to usual care were analyzed using an ANCOVA with pre-measurement values as covariate for the predicting group factor and post-measurement values as outcome variable (Field et al., 2012). In case not all assumptions were met, Quade's non-parametric ANCOVA was used. To discover whether effects were substantive, partial eta-squared ($\eta_{\text{p}}^{2}$) effect sizes including 90% confidence intervals were calculated, according to recommendations for pilot trials (Lee et al., 2014). Because this pilot RCT is not adequately powered for all secondary outcomes, the interpretation of secondary outcomes focused on the effect size estimates, as recommended by Lee et al. (2014). Effect sizes were interpreted to be small (0.01 ≤ $\eta_{\text{p}}^{2}$ < 0.06), medium (0.06 ≤ $\eta_{\text{p}}^{2}$ < 0.14), or large ($\eta_{\text{p}}^{2}$ > 0.14) (Cohen, 1988).

Statistical analysis was done by PM after data collection was completed. No interim analysis was performed.

## 3. Results

### 3.1. Recruitment and participant flow

**Figure 4:**
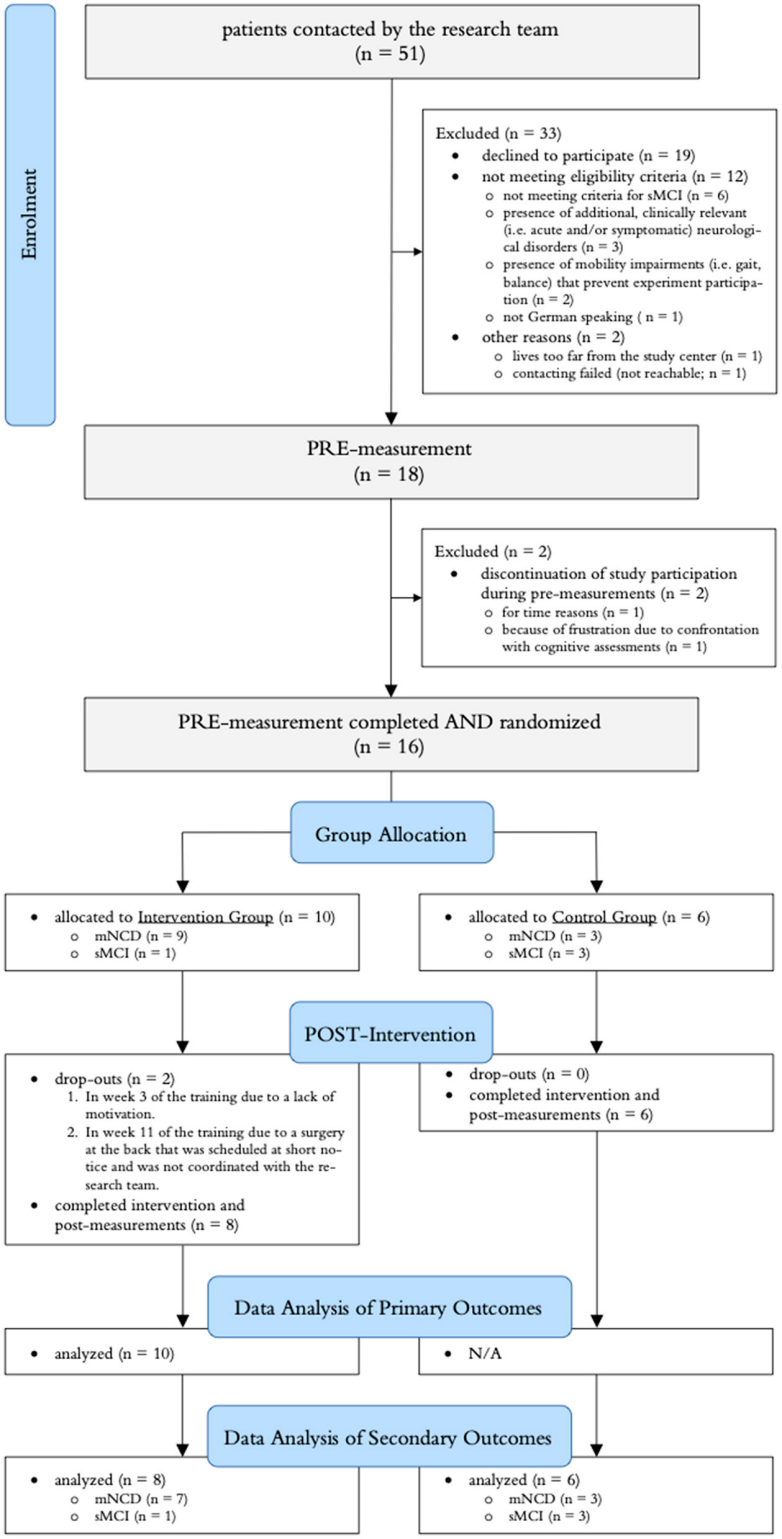
Summary of the participant flow throughout the study. mNCD, clinically diagnosed mild neurocognitive disorder; sMCI, screened for mild cognitive impairment.

A summary of the participant flow through the study is illustrated in Figure 4. Recruitment was stopped after the planned minimum sample size of 18 participants was reached. Of the 18 included participants, 13 were clinically diagnosed with mNCD and five fulfilled the criteria defined for sMCI. In the intervention group, nine participants started their training at home as planned and one participant was allowed to perform the training at the study center (ETH Hönggerberg) using the “Senso” because there was not enough space for the exergame device at the participants' home. Three minor adverse events (falls in participants' homes with bruises, but no more serious injuries) were recorded, all of which occurred in the intervention group (in two different participants, one of whom has mild frontotemporal NCD). All AEs were unrelated to the “Brain-IT” training.

### 3.2. Baseline data

Demographic characteristics of participants are summarized in Table 4. There were no significant between-group differences.

**Table 4:** Demographic characteristics of the study population.

	**Group: exergame (*****n*** = **10)**		**Group: usual care (*****n*** = **6)**		**Between-group difference**	
	**Mean**	**SD**	**Mean**	**SD**	**Test statistics** ^a^	***p*** **-value** ^b^
Age [years]	79.9	7.6	73.7	12.9	t_(7.14)_ = 1.076	0.317
Sex [number of females (%)]	3 (30.0)	N/A	4 (66.7)	N/A	N/A	0.302
Education [years]	13.2	4.2	13.2	1.2	t_(11.13)_ = 0.024	0.982
Body mass index [kg·m^−2^]	23.1	2.2	26.4	3.2	t_(7.92)_ = −2.265	0.054
Physical activity [min/week]	233.0	200.7	425.0	253.4	t_(8.78)_ = −1.582	0.149
**Etiological subtype:**						
mNCD due to Alzheimer's Disease	*n =* 7 (70%)		*n =* 4 (66.6%)			1.000
mild frontotemporal NCD	*n =* 1 (10%)		*n =* 0 (0%)			1.000
mNCD with Lewy Bodies	*n =* 0 (0%)		*n =* 0 (0%)			1.000
mild vascular NCD	*n =* 2 (20%)		*n =* 2 (33.3%)			0.604

a Statistics for the between-group differences tested with an independent t-test or Mann–Whitney U-test in case the data are not normally distributed;

b p-values for the between-group differences tested with an independent t-test or Mann–Whitney U-test in case the data are not normally distributed, or Fisher's exact test for categorical variables.

Abbreviations: SD, Standard Deviation.

### 3.3. Delivery of the interventions

#### 3.3.1. Type of usual care activities

For participants who completed the study, 75% of participants in the intervention group and 83% of participants in the control group reported that they received one or more structured or guided usual care activities(s) during study participation. Details on types of usual care activities are summarized in Table 5. Additionally, one participant in the intervention group had a stationary rehabilitation stay for 3 weeks focusing on gait and balance due to polyneuropathy. During the stay, the participant was able to continue with the “Brain-IT” training.

**Table 5:** Type of usual care activities.

**Type of usual care activities**	**Proportion of participants having received the respective intervention during study participation**		**Between-group difference**
	**Group: exergame (*****n** =* **8)**	**Group: usual care (*****n** =* **6)**	***p*** **-value** ^c^
Regular medication intake	*n =* 6 (75% of participants)	*n =* 4 (66.7% of participants)	1.000
Physiotherapy	*n =* 2 (25% of participants); median volume^b^ = 60 min/week	*n =* 1 (16.7% of participants); volume = 50 min/week	1.000
Occupational therapy	*n =* 1 (12.5% of participants); volume = 60 min/week	*n =* 0 (0% of participants)	1.000
Medical training therapy^a^	*n =* 2 (25% of participants); median volume = 60 min/week	*n =* 1 (16.7% of participants); volume = 75 min/week	1.000
(Computerized) cognitive training	*n =* 1 (12.5% of participants); volume = 30 min/week	*n =* 2 (33.3% of participants); median volume = 285 min/week	0.539

a Medical training therapy is prescribed by a doctor and guided and partly supervised by physiotherapists. It typically includes resistance, cardiorespiratory endurance, and balance exercises.

b Volume = time per training session [min] multiplied by the frequency of training [times/week].

c *p*-values for the between-group differences tested with Fisher's exact test for categorical variables.

#### 3.3.2. Actual delivery of the intervention

Participants who completed the training performed on average 54.4 ± 13.0 training sessions resulting in an average training volume of 1,128.3 ± 266.0 min over the 12-week intervention period. On average, 21.4 ± 1.1 training sessions were supervised by our study team. Average heart rates during the “facilitation,” “guidance,” and “coherence” phases were 96.9 ± 8.4 bpm, 86.2 ± 5.9 bpm, and 83.5 ± 5.9 bpm, respectively. No relevant deviations from the “Brain-IT” training concept were reported.

### 3.4. Primary outcomes

#### 3.4.1. Feasibility

**Figure 5:**
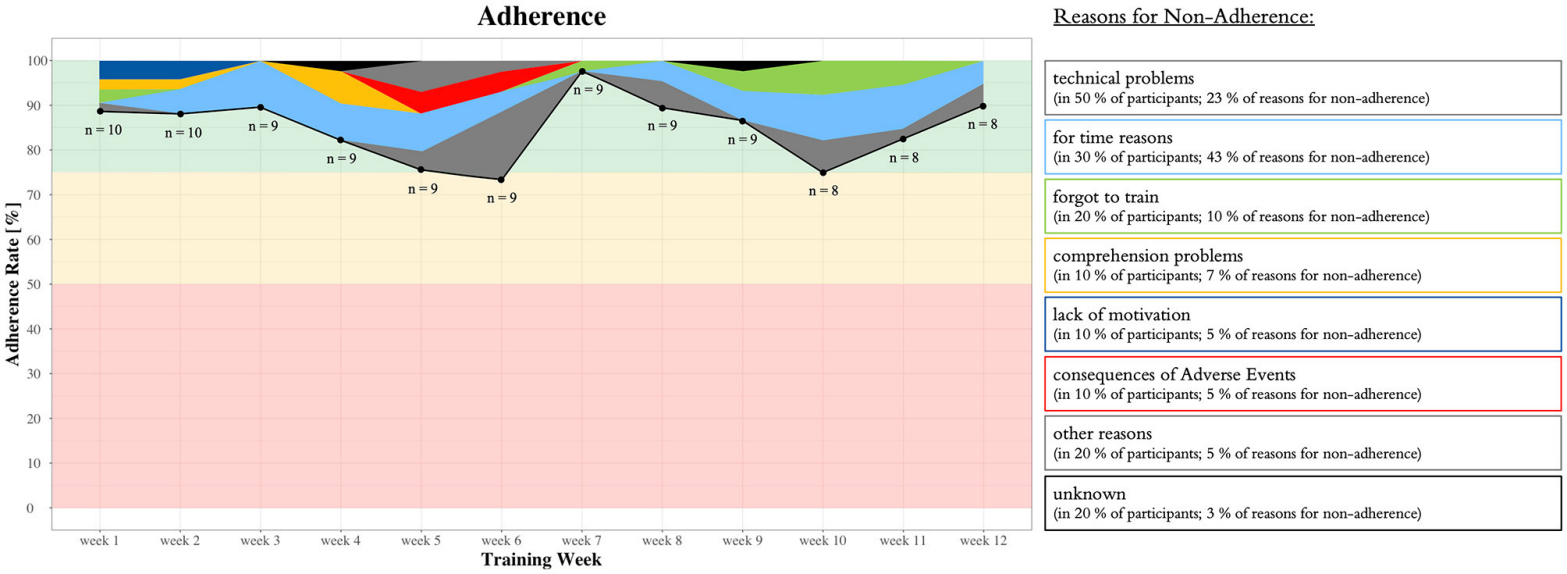
Detailed information on weekly adherence including the type and proportions of reasons for non-adherence as well as the predefined traffic light system with quantitative thresholds as guidelines to judge feasibility indicated in red, orange, and green (see the Section Feasibility or Table 1).

The first participant was contacted on 2 July 2021. The 18th participant was included on 11 March 2022. This results in an absolute recruitment rate of 2.2 participants per month. Out of the 51 individuals contacted by the study team, 18 were included in the study. This results in a relative recruitment rate of 35.3%. Two dropouts occurred in the intervention group, resulting in an attrition rate of 20%. The mean adherence rate to the training was 85.0 ± 21.4%. Detailed information on weekly adherence including the type and proportions of reasons for non-adherence is illustrated in Figure 5. “Other reasons” for non-adherence included organizational challenges (e.g., one participant went into a stationary clinic for 3 weeks and the training equipment first had to be transported to the clinic for the participant to be able to continue training).

The mean compliance rate to the training was 84.1 ± 21.6%. In total, 13 training sessions were started but not completed. Of these, reasons for non-compliance included (1) accidentally stopping the training by staying on the back plate of the exergame device for too long (in 40% of participants; 71% of reasons for non-compliance), (2) technical problems (in 20% of participants; 21% of reasons for non-compliance), or (3) unknown (in 10% of participants; 8% of reasons for non-compliance).

#### 3.4.2. Usability

**Figure 6:**
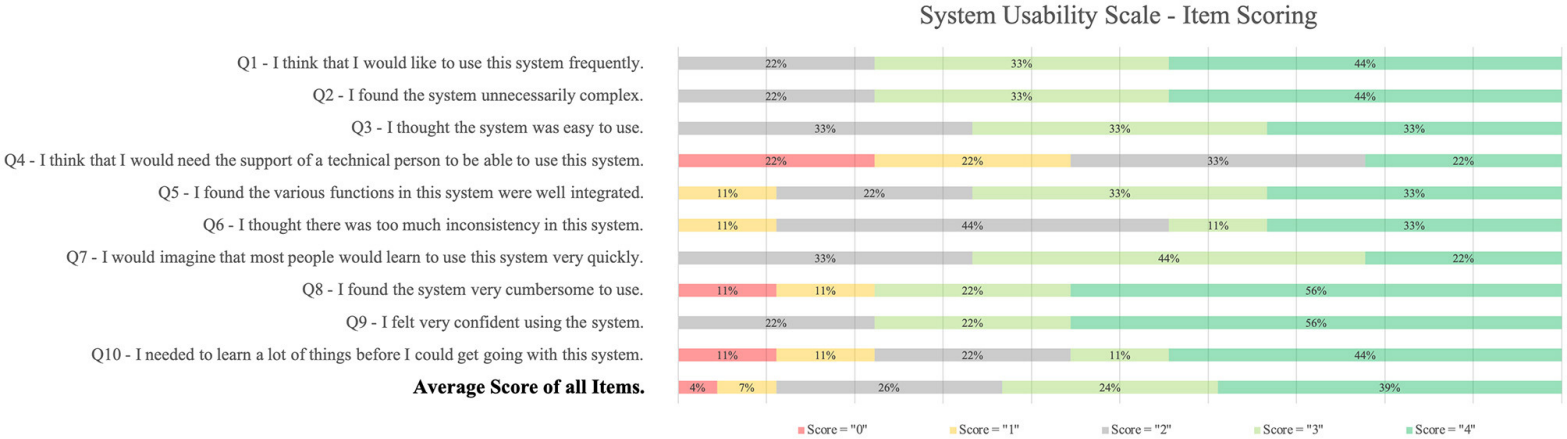
Details on item scoring of the system usability scale.

The mean system usability score was 71.7 ± 15.4. Details on item scoring are illustrated in Figure 6. The highest score was reached in question nine (“I felt confident using the system,” mea*n* = 3.3 points). The lowest score was reached in question four (“I think that I would need the support of a technical person to be able to use this system,” mea*n* = 1.8 points).

#### 3.4.3. Acceptance

**Figure 7:**
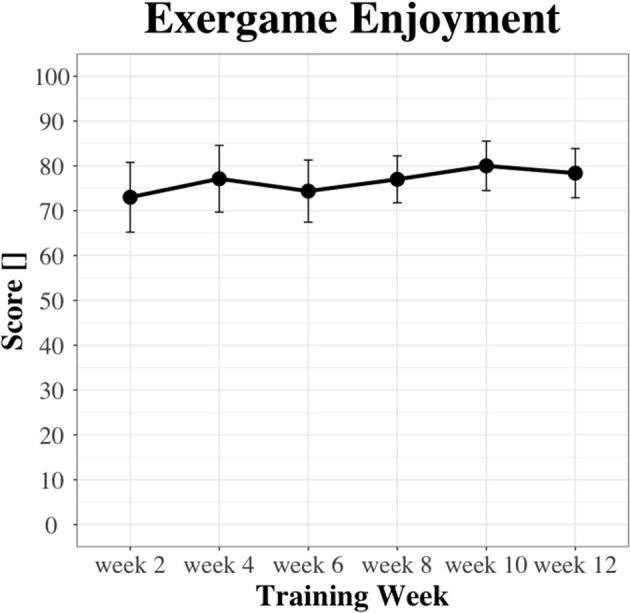
Biweekly scores on exergame enjoyment.

Biweekly scores on exergame enjoyment are illustrated in Figure 7. There was no main effect of time on exergame enjoyment [$\chi_{\left(5\right)}^{2}$ = 8.52, *p* = 0.13]. Exergame enjoyment was rated significantly (*p* = 0.03) higher in week 12 (media*n* = 78.0) compared to week 2 (median = 72.0), with a large (r = 0.75) effect size.

**Figure 8:**
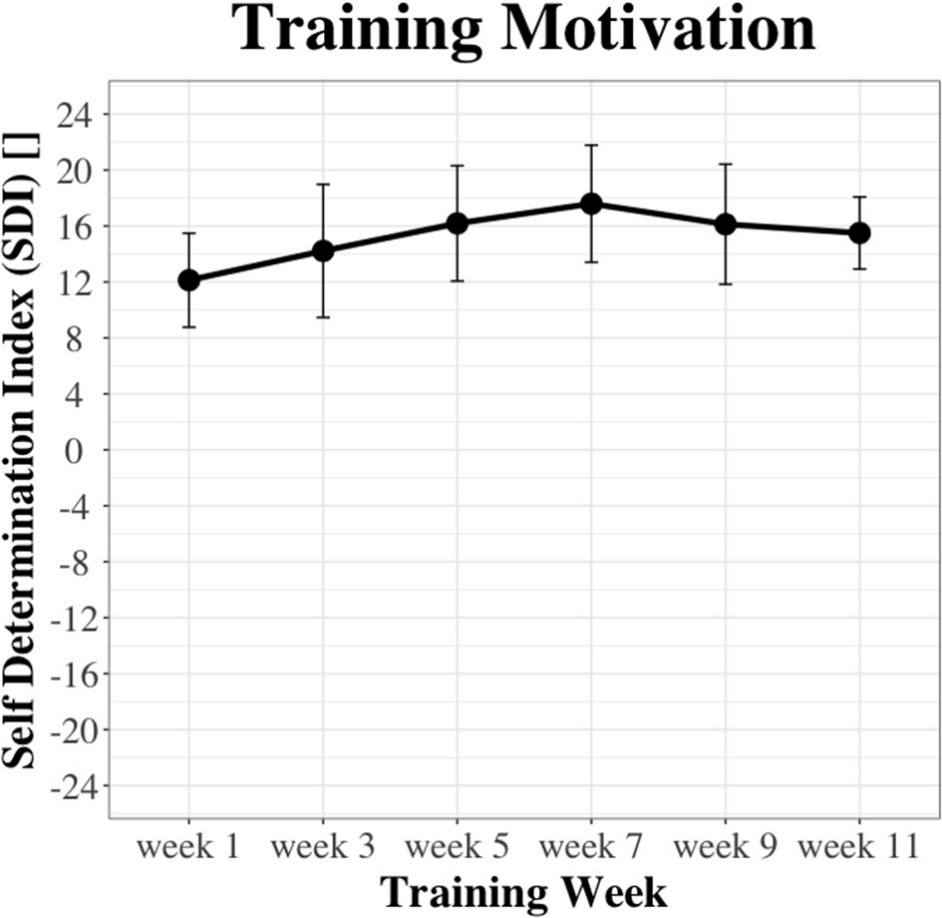
Biweekly scores on training motivation.

Biweekly scores on training motivation are illustrated in Figure 8. There was a significant effect of time on training motivation [$\chi_{\left(5\right)}^{2}$ = 11.31, *p* = 0.04]. The SDI was significantly (*p* = 0.03) higher in week 11 (median = 16.5) compared to week 1 (median = 12.38), with a large (r = 0.74) effect size.

According to the qualitative in-depth interviews, all participants reported to have perceived the training as useful. Six participants (75% of interviewed participants) described perceived changes in cognitive functioning, physical abilities, and/or wellbeing in response to training. Participants had difficulties describing the perceived changes. Those perceived changes that were described are summarized in Table 6.

**Table 6:** Summary of the perceived changes that were described by the participants in the semi-structured interviews.

	**Perceived changes described by the participants as a response to the question** ***“Do you feel any changes (e.g., mental and physical abilities, well-being) as a result of the training? If so, how exactly do these changes manifest themselves?”***	
	**Positive changes (perceived stabilization/ improvements)**	**Neutral/ negative changes (perceived (continued) deterioration)**
Cognitive functioning (overall)	f = 13, *n =* 5 (62.5% of participants)	f = 1, *n =* 1 (12.5% of participants)
Global cognition	f = 3, *n =* 3 (37.5% of participants)	No statements
Learning and memory	f = 1, *n =* 1 (12.5% of participants)	f = 1, *n =* 1 (12.5% of participants)
Complex attention	f = 6, *n =* 3 (37.5% of participants)	No statements
Executive function	f = 2, *n =* 1 (12.5% of participants)	No statements
Visuospatial skills	no statements	No statements
Social cognition	f = 1, *n =* 1 (12.5% of participants)	No statements
Language	No statements	No statements
Physical functioning	f = 3, *n =* 3 (37.5% of participants)	f = 1, *n =* 1 (12.5% of participants)
Coupling of brain–body functioning	f = 3, *n =* 2 (25% of participants)	No statements
Risk for falls	f = 1, *n =* 1 (12.5% of participants)	No statements
Fear of falling	f = 1, *n =* 1 (12.5% of participants)	No statements
Mood and wellbeing	f = 9, *n =* 3 (37.5% of participants)	No statements
Transfer effects to IADL	f = 3, *n =* 2 (25% of participants)	No statements

Perceived changes in cognitive functioning were classified into the key neurocognitive domains (as defined by Sachdev et al., 2014 in line with DSM-V; American Psychiatric Association, 2013). f, frequency of various statements; n, number of participants making a specific statement; IADL, instrumental activities of daily living.

P-84932328: “*It's difficult to describe because the physical and mental functions are always connected in the end. I feel improvements in both areas. If I can think better and faster, then I can also react faster physically. That is very important for me. I also notice that when I go for a walk and use public transport. I always have to anticipate and react very quickly. [...] That works better and faster. I even dare to overcome a certain height when I get off the bus. Earlier, I was terrified that I wouldn't make it and would fall. That also went better. I can generally adjust better to such situations. That is very important. Because I know: If I have my 17th fall, it's not good. I also see certain things more positively. Now I'm also happy about little things again and don't demonize everything that doesn't work or didn't work out.”*

P-3223376: “*I was always very cheerful after the training. So, it had a positive effect on my wellbeing, that's for sure.”*

Participants reported positive experiences with specific games [f = 7, *n* = 4 (50% of participants)] and that the training has brought some structure into everyday life [f = 2, *n* = 2 (25% of participants)]. The training dosage was perceived as good [f = 1, *n* = 1 (12.5% of participants)] and the training varied [f = 1, *n* = 1 (12.5% of participants)].

P-37740093: “*I found the game with the shopping list [game “Shopping Tour'] to be a good exercise to train the memory. Or also the one with the sounds [game ‘Simon’ and ‘Simon_numbered’]. [...] The training has also given a structure to everyday life, which was very good.”*

P-77422816: “*The game 'Habitats' was the one I liked the most. And in general, that the training allowed me to move. I like to move a lot. But the combination with the mind, that's actually what I liked most.”*

Fifty percent of participants would like to continue with the training as it is. The remaining participants would like to continue the training but only if it is effective (*n* = 1, 12.5% of participants) and/or if adjustments are made to the training or the exergame device (*n* = 4, 50% of participants). These adjustments include a drastic reduction in technical problems (*n* = 3, 37.5% of participants) and/or improvements in monitoring and individualized adaptation of task demands (*n* = 1, 12.5% of participants). None of the participants reported that they definitely did not want to continue the training.

P-68113192: “*I would have loved to continue the training. […] Of course, I would be willing to pay to use the system. I really believe that it helps me a lot and improves my quality of life. […] But because of the frustration caused by the technical problems, I don't want to continue. If it wasn't for the technical problems, I would have liked to continue. The structure and volume of the training itself was good and the training was varied.”*

P-05558066: “*Yes, I would like to continue the training. But you have to be able to measure better whether the performance remains constant or whether you improve or deteriorate a little. It has to be measurable. There was just too much that didn't work [technically].”*

For the training to be optimal, participants reported that technical problems must be solved [f = 4, *n* = 3 (37.5% of participants)], that changes in performance over time should be regularly discussed [f = 2, *n* = 2 (25% of participant)], and that individualized adaptation of task demands is improved [f = 2, *n* = 2 (25% of participant)].

P-53458467: “*It would be beneficial to discuss the results with a professional every week. I partly had the feeling that it was miserable again today and of course it would be nice to hear if it was the opposite. These discussions would help to know how things are going and whether my memory has progressed or rather regressed.”*

Additionally, one participant suggested that the effort required to start up the system and start training should be reduced. Finally, one participant suggested specific adaptations of existing games, and three participants suggested that new and/or additional games should be offered.

### 3.5. Secondary outcomes

The results of the “TAP Incompatibility” were excluded from analysis because many participants had comprehension problems, which led to invalid results. The remaining results of secondary endpoints are summarized in Table 7 in detail. In short, the intervention group improved their score in global cognitive functioning from 52.8 ± 11.7 points at pre-measurements to 59.8 ± 11.0 points at post-measurements, while the control group showed a decline from 60.1 ± 8.0 points to 58.9 ± 6.7 points. There was a medium, but non-significant effect [F_(1, 11)_ = 0.96, *p* = 0.35, η^2^_*p*_ = 0.080], in favor of the intervention group. Regarding domain-specific cognitive functioning, there were small favorable effects on learning and memory and visuospatial skills in favor of the intervention group, small-to-medium favorable effects on executive functioning in favor of the intervention group, and mixed findings on effects on complex attention. Regarding resting-state cortical activity, participants in the intervention group showed changes in the direction of the brain functions of HOAs in the post-intervention EEG measurements when using beta amplitude power and alpha phase synchrony as analysis methods. Regarding spatiotemporal parameters of gait, there were no relevant between-group effects on walking speed and stride length, although walking speed decreased within the intervention group. There was a moderate effect on stride duration in favor of the control group and small-to-moderate favorable effects on gait parameters that indicate a more stable gait (i.e., an increase in swing time and single support time, and a decrease in stance phase duration and double support time) in favor of the intervention group. Regarding psychosocial factors, there was a small favorable effect on quality of life and depression in favor of the intervention group, a moderate favorable effect on stress in favor of the intervention group, and no relevant between-group effects on anxiety. Regarding resting cardiac autonomic regulation, there were no relevant between-group effects. None of the effects were statistically significant.

**Table 7:** Statistics for all secondary outcomes.

**Outcome:**	**Check of assumptions and type of analysis:**		**Group: exergame**			**Group: usual care**			**ANCOVA statistics:**		
			**PRE-measurement**	**POST-measurement**	**sample**	**PRE-measurement**	**POST-measurement**	**Sample**			
	**All assumptions for parametric analysis met?**	**Type of** **analysis**	**Mean** ±**SD or median (IQR)**	**Mean** ±**SD or** **median (IQR)**	**n**	**Mean** ±**SD or** **median (IQR)**	**Mean** ±**SD or median (IQR)**	**n**	***p*** **-Value**	**F-Value**	$\eta_{\text{p}}^{2}$ **[90% CI]**
**Part 1—Cognitive Functioning**											
1.1 Global Cognition											
Qmci total score []	✓	parametric	52.8 ± 11.7	59.8 ± 11.0	8	60.1 ± 8.0	58.9 ± 6.7	6	0.348	0.961	0.080 [0, 0.348]
1.2 Learning and memory											
WMS-IV-LM score part 1 []	x	Non-parametric	26.0 (21.0)	30.0 (17.0)	8	24.0 (6.0)	26.0 (3.5)	6	0.646	0.223	0.020 [0, 0.246]
WMS-IV-LM score part 2 []	x	Non-parametric	6.0 (12.5)	6.5 (8.5)	8	9.5 (4.8)	5.5 (14.5)	6	0.843	0.041	0.004 [0, 0.152]
WMS-IV-LM score part 2—Recognition []	✓	Parametric	15.6 ± 3.7	15.9 ± 3.4	8	17.7 ± 3.3	17.6 ± 2.0	6	0.738	0.117	0.011 [0, 0.210]
DSF total score []	x	Non-parametric	5.0 (2.3)	7.0 (1.5)	6^a^^,^^b^	8.0 (1.5)	7.5 (2.5)	6	0.992	0.000	0.000 [0, 0.000]
DSF maximal span []	✓	Parametric	3.8 ± 1.3	5.2 ± 1.3	6^a^^,^^b^	5.8 ± 1.5	5.2 ± 1.5	6	0.830	0.049	0.005 [0, 0.162]
1.3 Complex attention											
TMT-A—Completion Time [s]	x	Non-parametric	38.0 (26.1)	39.4 (6.1)	7^b^	45.66 (10.7)	39.0 (16.4)	6	0.370	0.882	0.081 [0, 0.341]
TMT-A—Number of Errors []	x	Non-parametric	1.0 (3.0)	1.0 (3.5)	7^b^	1.0 (1.5)	1.5 (2.5)	6	0.426	0.690	0.065 [0, 0.321]
TAP Alertness (Condition A)—RT [ms]	x	Non-parametric	310.0 (165.0)	373.8 (191.9)	8	261.5 (51.5)	243.2 (34.5)	6	0.155	2.330	0.175 [0, 0.444]
TAP Alertness (Condition B)—RT [ms]	x	Non-parametric	299.5 (148.8)	355.2 (111.6)	8	277.5 (66.1)	256.8 (56.2)	6	0.093	3.377	0.235 [0, 0.494]
TAP Go-NoGo—RT [ms]	x	Non-parametric	456.5 (181.8)	566.9 (162.0)	8	430.0 (63.0)	423.5 (77.7)	6	0.082	3.671	0.250 [0, 0.506]
TAP Go-NoGo—Number of Errors []	✓	Parametric	3.3 ± 2.4	2.1 ± 2.0	8	2.2 ± 1.9	3.0 ± 1.3	6	0.223	1.672	0.132 [0, 0.404]
1.4 Executive Functioning											
HOTAP-A Combi-Score [points/min]	✓	Parametric	3.9 ± 2.0	4.7 ± 2.5	8	5.4 ± 1.8	5.8 ± 1.7	6	0.939	0.006	0.001 [0, 0.030]
DSB Total Score []	x	Non-parametric	4.5 (3.3)	4.3 (1.5)	6^a^^,^^b^	6.0 (2.3)	6.0 (2.3)	6	0.998	0.000	0.000 [0, 0.000]
DSB Maximal Span []	x	Non-parametric	3.5 (1.0)	4.0 (0.8)	6^a^^,^^b^	4.6 (1.8)	4.0 (0.8)	6	0.387	0.827	0.084 [0, 0.335]
TMT-B—Completion Time [s]	x	Non-parametric	185.9 (124.8)	111.8 (107.9)	7^b^	86.4 (47.3)	66.6 (23.7)	6	0.362	0.913	0.084 [0, 0.344]
TMT-B—Number of Errors []	x	Non-parametric	7.0 (16.0)	2.0 (9.0)	7^b^	5.0 (9.0)	1.5 (3.3)	6	0.952	0.004	0.000 [0, 0.000]
1.5 Visuospatial Skills											
MRT—RTs [ms]	x	Non-parametric	3,471 (1,879)	3,082 (601)	7^b^	4,431 (2,737)	3,653 (1,918)	6	0.187	2.009	0.167 [0, 0.426]
MRT—Score []	✓	Parametric	45.0 + 8.7	49.1 ± 9.1	7^b^	44.8 ± 11.9	44.7 ± 11.1	6	0.182	2.054	0.170 [0, 0.428]
**Part 2—EEG**											
2.1 Amplitude Power:											
Beta (13–30 Hz) power of Cz [uV^2^/Hz]	✓	Parametric	0.72 ± 0.44	0.42 ± 0.41	4^c^^,^^d^	0.34 ± 0.24	0.81 ± 0.31	4^c^	0.384	0.911	0.154 [0, 0.379]
Theta (4–8 Hz) power of T7 [uV^2^/Hz]	x	Non-parametric	0.36 (0.24)	0.87 (0.19)	5^c^^,^^d^	0.54 (0.18)	0.84 (1.21)	4^c^	0.828	0.052	0.009 [0, 0.165]
Theta (4–8 Hz) power of T8 [uV^2^/Hz]	x	Non-parametric	0.33 (0.16)	0.92 (0.32)	5^c^^,^^d^	0.32 (0.35)	0.36 (1.88)	4^c^	0.397	0.833	0.122 [0, 0.336]
Theta (4–8 Hz) power of FT7 [uV^2^/Hz]	x	Non-parametric	0.31 (0.42)	0.68 (0.67)	6^c^	0.40 (0.38)	0.36 (0.71)	4^c^	0.337	1.062	0.132 [0, 0.358]
Theta (4–8 Hz) power of FT8 [uV^2^/Hz]	x	Non-parametric	0.37 (0.52)	1.28 (1.69)	6^c^	0.22 (0.25)	0.07 (0.30)	3^(3, 4)^	0.075	4.632	0.436 [0, 0.541]
2.2 Phase Synchrony:											
Alpha (8–13 Hz) Fp2-C4 []	x	Non-parametric	0.41 (0.04)	0.42 (0.04)	6^c^	0.43 (0.01)	0.42 (0.02)	4^c^	0.762	0.099	0.014 [0, 0.201]
Alpha (8–13 Hz) F7—T6 []	x	Non-parametric	0.42 (0.02)	0.43 (0.04)	6^c^	0.42 (0.11)	0.46 (0.19)	4^c^	0.337	1.060	0.132 [0, 0.357]
Alpha (8–13 Hz) T3—T6 []	x	Non-parametric	0.44 (0.39)	0.71 (0.59)	6^c^	0.43 (0.02)	0.46 (0.06)	4^c^	0.596	0.309	0.042 [0, 0.265]
Alpha (8–13 Hz) T5—T6 []	x	Non-parametric	0.42 (0.03)	0.43 (0.44)	6^c^	0.43 (0.12)	0.43 (0.16)	4^c^	0.540	0.414	0.056 [0, 0.284]
**Part 3—Gait**											
Walking Speed [m · s^−1^]	x	Non-parametric	1.22 (0.26)	1.00 (0.27)	7^c^	1.15 (0.50)	1.15 (0.30)	6	0.964	0.002	0.000 [0, 0.000]
Stride Duration [ms]	x	Non-parametric	1,070 (195)	1,130 (155)	7^c^	1,040 (120)	1,045 (88)	6	0.289	1.254	0.111 [0, 0.374]
Stride Length [cm]	x	Non-parametric	123.0 (9.0)	118.0 (18.5)	7^c^	116.0 (36.3)	114.5 (30.0)	6	0.905	0.015	0.002 [0, 0.091]
Stance Phase Duration [% stride duration]	✓	Parametric	61.0 ± 3.0	61.0 ± 2.1	8	60.3 ± 1.8	61.5 ± 2.0	6	0.334	1.022	0.085 [0, 0.354]
Swing Phase Duration [% stride duration]	✓	Parametric	39.0 ± 3.0	39.0 ± 2.1	8	39.7 ± 1.8	38.5 ± 2.0	6	0.333	1.024	0.085 [0, 0.354]
Single Support Time [%]	✓	Parametric	39.2 ± 2.9	39.0 ± 2.0	8	39.6 ± 2.0	38.6 ± 2.1	6	0.499	0.488	0.042 [0, 0.295]
Double Support Time [%]	✓	Parametric	10.8 ± 2.9	10.9 ± 2.0	8	10.3 ± 1.9	11.5 ± 2.0	6	0.370	0.875	0.074 [0, 0.340]
**Part 4—Psychosocial Factors**											
Quality of Life (QoL-AD) []	x	Non-parametric	36.0 (5.8)	36.5 (10.3)	8	36.5 (7.3)	36.0 (3.0)	6	0.746	0.110	0.010 [0, 0.206]
DASS-21—Depression []	x	Non-parametric	4.5 (2.5)	2.5 (4.5)	8	3.0 (5.0)	3.0 (4.8)	6	0.822	0.053	0.005 [0, 0.167]
DASS-21—Anxiety []	x	Non-parametric	2.5 (2.8)	1.0 (1.25)	8	1.0 (0.75)	1.0 (1.5)	6	0.919	0.011	0.001 [0, 0.069]
DASS-21—Stress []	x	Non-parametric	5.0 (4.5)	4.0 (3.5)	8	3.0 (0.75)	4.5 (3.25)	6	0.335	1.016	0.085 [0, 0.353]
**Part 5—heart rate variability**											
mRR [ms]	x	Non-parametric	804.0 (216.5)	737.0 (217.7)	6^c^	852.0 (136.0)	797.0 (100.0)	5^c^	0.508	0.481	0.057 [0, 0.294]
RMSSD [ms]	x	Non-parametric	27.1 (40.0)	25.2 (41.5)	6^c^	8.3 (6.0)	6.1 (7.2)	5^c^	0.789	0.076	0.009 [0, 0.186]
pNN50 [%]	x	Non-parametric	4.6 (37.4)	9.7 (26.5)	6^c^	0.0 (0.0)	0.0 (0.0)	5^c^	0.928	0.009	0.001 [0, 0.054]
HF [ms^2^]	x	Non-parametric	92.5 (793.6)	289.5 (557.3)	6^c^	23.0 (44.0)	9.0 (57.0)	5^c^	0.453	0.623	0.072 [0, 0.313]
HFnu [nu]	✓	Parametric	55.1 ± 23.0	39.4 ± 29.7	6^c^	66.2 ± 23.5	57.8 ± 18.1	5^c^	0.412	0.750	0.086 [0, 0.327]
SD1 [ms]	x	Non-parametric	19.2 (28.4)	17.85 (29.4)	6^c^	5.8 (4.3)	4.3 (5.1)	5^c^	0.789	0.076	0.009 [0, 0.186]
PNS-Index []	x	Non-parametric	0.02 (1.06)	−0.59 (1.65)	6^c^	−1.04 (1.0)	−1.35 (0.62)	5^c^	0.929	0.009	0.001 [0, 0.237]

The normal distribution of data was checked using the Shapiro–Wilk test and Q-Q plots. The level of significance was set to *p* ≤ 0.05 (two-sided, uncorrected). The assumption of homogeneity of variance was checked using Levene's test. In case all assumptions for analysis of covariance (ANCOVA) were met, effects of the addition of the “Brain-IT” training concept to usual care as compared to usual care were analyzed using an ANCOVA with the pre-measurement value as a covariate for the predicting group factor and the post-measurement value as outcome variable (Field et al., 2012). In case not all assumptions were met, Quade's non-parametric ANCOVA was used. To discover whether effects are substantive, partial eta-squared ($\eta_{\text{p}}^{2}$) effect sizes were calculated for all primary and secondary outcomes. Effect sizes were interpreted to be small (0.01 ≤ $\eta_{\text{p}}^{2}$ < 0.06), medium (0.06 ≤ $\eta_{\text{p}}^{2}$ < 0.14), or large ($\eta_{\text{p}}^{2}$ > 0.14) (Cohen, 1988).

a Missing data due to comprehension problems of the test;

b missing data because the measurement had to be stopped due to attentional exhaustion of the participant;

c missing data due to technical problems with the measurement device;

d missing data due to insufficient data quality. Qmci, Quick Mild Cognitive Impairment Screen; WMS-IV-LM, subtest “logical memory” of the Wechsler Memory Scale- fourth edition; PEBL, Psychology experiment building language; DSF, Digit Span Forward; DSB, Digit Span Backward; TMT-A and B, Trail Making Test Part A and B; TAP Alertness, subtest “Alertness” of the Test of Attentional Performance; TAP Go-NoGo, subtest “Go-NoGo” of the Test of Attentional Performance; TAP Incompatibility, subtest “Incompatibility” of the Test of Attentional Performance; HOTAP-A, HOTAP picture-sorting test part A; MRT, Mental Rotation Task; MRI, magnetic resonance imaging; IADL, Instrumental Activities of Daily Living; QOL-AD, Quality of Life-Alzheimer's Disease; DASS-21, Depression, Anxiety and Stress Scale-21; vm-HRV, vagally mediated heart rate variability; SD, standard deviation; IQR, interquartile range; n, sample size; ANCOVA, analysis of covariance; $\eta_{\text{p}}^{2}$ [90% CI], partial eta-squared [90% confidence interval].

## 4. Discussion

This study evaluated the feasibility, system usability, and acceptance of the “Brain-IT” project and the “Brain-IT” training concept—a newly developed training concept combining exergame-based motor–cognitive training and heart rate variability (HRV)-guided resonance breathing for the secondary prevention of mNCD. The results suggest that (1) the “Brain-IT” project is feasible (with amendments to the study protocol that allow increasing the absolute recruitment rate); (2) the “Brain-IT” training is feasible for older adults who have mNCD, indicated by acceptable adherence, compliance, and attrition rates. However, frequent occurrences of technical problems and difficulties in using the “Senso Flex” training system were identified as barriers to performing the “Brain-IT” training; (3) the “Senso Flex” is usable as a means to implement the “Brain-IT” training concept for older adults who have mNCD, indicated by an acceptable mean system usability score; (4) the “Brain-IT” training was well-accepted by older adults who have mNCD, indicated by a high level of exergame enjoyment, increases in exergame enjoyment and internalization of training motivation with large effect sizes from the first to the last measurement, and an acceptable perceived usefulness; and (5) preliminary data on the effects of the “Brain-IT” training are promising.

### 4.1. Feasibility

#### 4.1.1. Recruitment

Our main difficulty in recruitment was the ability to find reliable and committed clinical collaboration partners. This may be explained by the highly competitive nature of research with individuals who have mNCD in Switzerland and/or the COVID-19 pandemic. As a result, not enough individuals could be reached out to, although the number of individuals who have m-MNCD in Switzerland is high (Alzheimer Europe, 2019). Many of the individuals contacted by the study team participated in the study. Our relative recruitment rate is above average when compared to the literature [i.e., a median recruitment rate of 26% (range: 3.4–59%) was determined for exergame-based training in individuals who have mNCD in the studies analyzed in Zhao et al., 2020]. This suggests that our study protocol is feasible without further adjustments with regard to the relative recruitment rate.

#### 4.1.2. Adherence and compliance

Despite their conservative calculation, the adherence and compliance rates found in this study were higher compared to the average pooled adherence rates of comparable intervention studies (Panza et al., 2018; Di Lorito et al., 2020; Zhao et al., 2020; Swinnen et al., 2022). Previous (pilot) RCTs investigating exergame-based training in older adults who have mNCD on average found slightly lower adherence and compliance rates. In particular, adherence rates of 78% (approximated by the reported average number of training sessions per week divided by the target training frequency of 5x/week) (Anderson-Hanley et al., 2018a) and 79.2% (calculated by dividing the number of played sessions vs. the number of planned sessions) (Robert et al., 2021) and compliance rates of 88.5% (calculated by dividing total play duration vs. planned minimal training time) (Robert et al., 2021) and 55.5% (calculated from reported exercise time divided by the defined activity goal) (Padala et al., 2012) were reported. The remaining studies reported training adherence (Delbroek et al., 2017; Mrakic-Sposta et al., 2018; Amjad et al., 2019) or compliance (Delbroek et al., 2017; Anderson-Hanley et al., 2018a; Mrakic-Sposta et al., 2018; Amjad et al., 2019) insufficiently. Overall, our results are in line with previous findings that adherence to exergame-based training is typically high in older adults who have m-MNCD (Zhao et al., 2020; Swinnen et al., 2022).

Most previous exergame-based (Anderson-Hanley et al., 2018a; Robert et al., 2021) or conventional (Donnezan et al., 2018; Panza et al., 2018; Di Lorito et al., 2020) training studies including older adults who have mNCD and reporting adherence prescribed one-on-one supervision of training (Donnezan et al., 2018; Panza et al., 2018; Di Lorito et al., 2020), or did not report supervision (Anderson-Hanley et al., 2018a; Robert et al., 2021), opposed to that we expected our participants to train partly independently. Therefore, participants had to remember and motivate themselves to do their training. They managed to do this extremely well, as adherence rates stayed high throughout the intervention period with gradually reduced supervision over time. This is promising because home-based and partly unsupervised training allows a time and cost-efficient way of training. Furthermore, it is preferred by older adults who have mNCD (Manser et al., 2023a) and older adults in general (Yardley et al., 2008; Valenzuela et al., 2018), and reduces barriers to exercise (Valenzuela et al., 2018; Manser et al., 2023a). Next to a lack of time and inability to travel to the training facility, difficulties in using technology are among the most prevalent reasons for discontinuing technology-based training programs (Valenzuela et al., 2018). Our good results in training adherence and compliance might also be because the “Brain-IT” training concept with some of the exergames included in the training was purpose-developed specifically for older adults who have mNCD guided by the MIDE-Framework (Manser and Bruin, 2020, 2021). As reported in more detail in the “Brain-IT” training concept (Manser and Bruin, 2021), the training concept included individual supervision (including telephone support of the study team in case of technical difficulties or comprehension problems), a familiarization phase of 2 weeks, was individually tailored, included visual and auditory feedback in real-time to enrich the game experience, and was designed to support to overcome known exercise barriers. Additionally, each participant was provided with an individually adapted training manual. All these elements are support strategies with theoretical underpinnings (programs based on behavior change theories) that may help promote training adherence and should therefore be considered when planning training concepts in older adults who have mNCD (van der Wardt et al., 2017).

In the following iterative research step, further improvements regarding training adherence and compliance should be considered. Fifty percent of participants experienced technical problems (e.g., network errors, software problems, or difficulties in handling the exergame device), ranking as the second most common reason for non-adherence. Non-compliance was entirely explained by technical problems. An additional potentially preventable reason for non-adherence was comprehension problems at the beginning of the “Brain-IT” training. These two issues have been identified in a qualitative study conducted in the first phase of the “Brain-IT” project as key issues as well (Manser and Bruin, 2021; Manser et al., 2023a). Details of all identified issues together with suggestions on how to resolve these were communicated to the company providing the “Senso Flex” after completing the qualitative study and again after completing this study. So far, we are unaware whether the technical problems and difficulties in handling the device were addressed. Importantly, we only reported technical problems that hindered participants from starting a training session in non-adherence. Occurrence of technical problems during training, however, was far more common and mainly included problems with the sensitivity of the sensors hindering interaction with the device and orientation problems on the device (i.e., participants unintentionally leave the middle plate because it is too small, is not properly marked on the “Senso Flex,” and participants do not notice the feedback to return to the middle plate on the screen). These technical problems did not result in early termination of the training session in most cases; however, they did lead to frustration among participants. This negatively influenced the will to continue using the system in future in some participants. Regarding the second key issue of comprehension problems, up to now, an instructional text is displayed before starting each game. However, individuals who have mNCD often have difficulties understanding written instructions and transferring these to the actual tasks. Therefore, more patient-friendly instructions (e.g., step-by-step video instructions or interactive “trial-run” instructions that combine visual and verbal instructions) should be offered (Manser et al., 2023a). As the company providing the “Senso Flex” has been unable to change this item, we have alternatively focused on practical demonstrations and provided each participant a detailed training manual that complements instructions provided by the exergame device. This solution is suboptimal because it requires participants to switch between the exergame device and the training manual while the training manual does not allow to offer the described more patient-friendly types of instructions. Future iterations should examine whether alternative hardware and software solutions provide more feasible options to implement the “Brain-IT” training concept. To facilitate this process, we will modify the “Brain-IT” training concept and add specific information that allows the training concept to be adapted to other hardware and software solutions. A possible solution to circumvent some of the technical problems would be the consideration of alternative peripherals. Recent research shows an increased use of camera-based systems and virtual or augmented reality headsets, which offer a wealth of new possibilities for optimizing these interventions (López-Nava et al., 2023).

#### 4.1.3. Attrition

The attrition rate found in this study was similar to the average pooled attrition rate of comparable intervention studies (Di Lorito et al., 2020; Swinnen et al., 2022). Previous (pilot) RCTs investigating exergame-based training in older adults who have mNCD also found similar attrition rates of 9% (Padala et al., 2012; Amjad et al., 2019), 20% (Delbroek et al., 2017; Mrakic-Sposta et al., 2018), and 55% (Anderson-Hanley et al., 2018b) or reported attrition insufficiently (Robert et al., 2021). Reasons for discontinuing the training and/or dropping out in these studies included time reasons (Anderson-Hanley et al., 2018b), inappropriate task difficulty (Anderson-Hanley et al., 2018b), voluntary withdrawal (Padala et al., 2012), medical conditions unrelated to the training (Delbroek et al., 2017; Anderson-Hanley et al., 2018b), or reasons independent from the training (Mrakic-Sposta et al., 2018). In our study, similar reasons for dropping out were reported. One of the dropouts in our study would have been preventable with better communication with the recruitment partner. The respective recruiting partner was informed, and measures were taken to improve communication between recruiting partners and the study team.

### 4.2. Usability

We found a considerably lower system usability score compared to a similar pilot study with older people that found a mean SUS score of 83.6 ± 13.7 points. The latter study differed regarding participants' lower mean age (73.0 years), not having any cognitive impairment, and using the “Senso” instead of the “Senso Flex.” Consistent with the results of this study, the lowest score was found in question four (Altorfer et al., 2021). The fact that a substantial proportion of our participants reported needing technical support to use the system in our study is problematic for a home-based training system aimed to be used (partly) independently. This needs to be addressed in further iterative development steps. Study investigators supervising participants indicated that the need for technical support stems from the effort required to start up the system and start training and in the occurrence of technical problems. This is mirrored in the feedback of one participant in the semi-structured interviews and through the reported technical difficulties and comprehension problems. These issues were anticipated based on the results of our qualitative study (Manser et al., 2023a). To overcome these anticipated issues, we implemented support strategies specifically for the participants in the training group (as described in the Section Participant retention). Although these strategies were experienced as helpful by participants and should, therefore, be maintained in future studies, some further support should be considered. Because technical problems are in general overwhelming to individuals who have mNCD (Manser et al., 2023a), they need to be drastically reduced to improve system usability. This would potentially reduce participants' dependence on study personnel. Additionally, providing more patient-friendly instructions might help to reduce comprehension problems discussed in the Section Adherence and compliance.

### 4.3. Acceptance

Our findings on acceptance of our training are consistent with previous research showing that exergames may increase or enhance motivation to engage in rehabilitation activities (Zhao et al., 2020). Motivation (especially intrinsic motivation) is a key factor for promoting positive behavioral changes (Ryan and Deci, 2000) (e.g., adherence to exercise) in older adults with or without cognitive impairment (Teixeira et al., 2012; Devereux-Fitzgerald et al., 2016; Di Lorito et al., 2019; Behzadnia et al., 2020; Collado-Mateo et al., 2021). More autonomous forms of motivation refer to engagement in a task based on intrinsic motivators such as exercise enjoyment or personal importance to perform the exercises (Ryan and Deci, 2000). Intrinsic motivation of individuals who have mNCD can be mainly promoted by excitement, enjoyment, or fun at exergaming. These factors can be supported through specific game components and the feeling of being optimally challenged. Additionally, individuals who have mNCD are motivated by the perceived effectiveness of training (Manser et al., 2023a). This is in line with findings for HOA, which showed that older adults were motivated by perceived health effects as well as the joy of playing exergames (Subramanian et al., 2020). Exercise enjoyment has been described as “*an optimal psychological state (i.e., flow) that leads to performing an activity primarily for its own sake and is associated with positive feeling state”* (Kimiecik and Harris, 1996). Based on our results, it seems fair to say that our interactive and participatory design and development process of the “Brain-IT” training concept (Manser and Bruin, 2021) resulted in an enjoyable training experience promoting internalization of training motivation and high levels of perceived usefulness. This observation might also explain the high levels of adherence to the training, because higher adherence rates to technology-based exercises may be largely explained by high levels of enjoyment (Valenzuela et al., 2018). However, despite the increase in motivation over time, we also observed a slight decrease in motivation after week 7. The study investigators who supervised participants indicated that this decline was mainly due to increasing frustration with technical problems. From participants' perspectives, solving the technical problems, improving the individualized adaptation of task demands, and adding a regular discussion of changes in performance are among the most important required modifications. A considerable proportion of participants would only like to continue training provided technical problems are drastically reduced, emphasizing the importance of addressing the issues.

Regarding improvements in the individualized adaptation of task demands, we relied on the exergame device's performance outcomes, as explained in detail in the “Brain-IT” training concept (Manser and Bruin, 2021). However, this did not always work properly with the device used. The system offers an internal progression algorithm that theoretically allows to individually progress task demands in real-time according to these performance outcomes. However, this algorithm has not (yet) been scientifically validated (Manser and Bruin, 2021) and was found unsuitable for individuals who have cognitive impairment (Manser et al., 2023a). Therefore, we relied on predefined progression rules based on visually analyzing performance curves, as described in more detail in the “Brain-IT” training concept (Manser and Bruin, 2021). This approach worked well for games that were newly developed or adapted within the “Brain-IT” project (mainly the games in the neurocognitive domain of learning and memory) because the games include precision parameters or provide a summed point score identical to validated cognitive assessments. For other games, we used mean reaction time [ms] to monitor performance over time as the other performance variables were found unsuitable (Manser and Bruin, 2021). However, reaction times were highly variable, making it difficult to visually read out a performance plateau. Future research should focus on more reliable parameters, preferably with a strong background in sports science or neuroscience. Because such parameters are not available for most of the games currently offered on the “Senso (Flex),” the games should either be adapted to meet these requirements or alternative hardware and software solutions should be developed and/or investigated to improve monitoring and individualized adaptation of task demands. Regarding a regular discussion of performance, further investigations are required to elaborate on the optimal solution for individuals who have mNCD. In our qualitative study, we have reported mixed findings on how to deal with performance feedback, because performance feedback can be motivating for some individuals whereas for others it may induce negative feelings by confronting them with their limitations. In case performance feedback is given (as is currently the case with the “Senso (Flex)” system), it is imperative that the program presents not just a performance curve, ideally depicted as a rolling average rather than individual performance scores, but also provides a reason or explanation of changes in performance over time (which the company offering the “Senso Flex” is not (yet) able to provide) (Manser et al., 2023a).

### 4.4. Effects of the training

The observed medium effect on global cognition is slightly higher compared with pooled evidence of exergame-based training in older adults who have mNCD on global cognition, which reported small (Gavelin et al., 2021)-to-medium (Wang et al., 2019) effects favoring the intervention. Additionally, the observed medium effect size is slightly higher compared with pooled evidence of simultaneous motor–cognitive training with reported small (Stanmore et al., 2017; Gavelin et al., 2021)-to-medium (Biazus-Sehn et al., 2020; Han et al., 2022) effect sizes. Most other secondary outcomes also point in the direction of favorable effects of the addition of the “Brain-IT” training to usual care. Although these preliminary data must be interpreted with caution due to the limitations of our study, these results are in line with the literature pointing to the direction that favorable effects are achievable in cognitive (Bamidis et al., 2015; Stanmore et al., 2017; Anderson-Hanley et al., 2018a; Amjad et al., 2019; Wang et al., 2019; Zhao et al., 2020; Di Lorito et al., 2022; Han et al., 2022), physical (Zhao et al., 2020; Di Lorito et al., 2022), and psychosocial (Di Lorito et al., 2022) functioning with exergame-based training in individuals who have mNCD. As a follow-up, the effectiveness of our “Brain-IT” training concept is currently being investigated in an RCT [clinicaltrials.gov (NCT05387057); see study protocol (Manser et al., 2023c)].

### 4.5. Limitations

The outcomes of this pilot RCT should be interpreted with caution considering the following limitations: First, the sample size was small. We stopped recruitment after reaching the planned minimum sample size of 18 participants because reaching out to potential study participants was the main difficulty in our study (as discussed in the Section Recruitment). Subsequently, there was one dropout in the intervention group at week 10 and one participant withdrew from the study during pre-measurement. As a result, the actual sample size is slightly below the recommended sample size for pilot or feasibility studies (Julious, 2005). However, at the time when we included the 18th participant, it was evident that all remaining feasibility outcomes exceeded the quantitative thresholds for acceptable feasibility. Therefore, we decided to stop recruitment, evaluate all results, revise our “Brain-IT” training concept as well as the study procedures, and start planning the next phase of our project (see Manser et al., 2023c in line with Manser and Bruin, 2021). Second, usual care activities were assessed by participants' self-report. To counteract possible biased information, the study team asked specific questions about whether participants engaged in typical usual care activities (as described in the Section Control group) and actively involved participants' proxies when collecting this information. Third, as part of usual care activities, it was only assessed whether participants had a regular intake of medications. No further details were collected because the effects of the “Brain-IT” training were only a secondary outcome of this study. However, for future studies investigating the effectiveness of the addition of the “Brain-IT” concept to usual care, details on medication intake (i.e., type and dosage of medication) as well as changes in medication during the study are required and will be assessed (Manser et al., 2023c). Fourth, all preliminary data on the effects of the addition of the “Brain-IT” training concept to usual care must be interpreted with caution because the statistical analysis for secondary outcomes was underpowered, groups were unbalanced, and the control group achieved better results in various tests during pre-measurements. In addition, because we investigated the addition of the “Brain-IT” training to usual care, and because usual care activities were provided by the (memory) clinics where the participants were recruited, we were not able to standardize contact times, which may have affected some of our findings.

## 5. Conclusion

The “Brain-IT” project is feasible provided the absolute recruitment rate can be increased in future studies. The feasibility and usability of the “Brain-IT” training concept implemented with the “Senso Flex” are acceptable. However, frequent occurrences of technical problems and difficulties in using the exergame training system were identified as barriers to performing the “Brain-IT” training. To optimize the feasibility of the “Brain-IT” training with the “Senso Flex” device, improvements in hardware and software are necessary. In particular, the occurrence of technical problems must be drastically reduced. The device's software should be adapted to provide more patient-friendly instructions and more reliable performance parameters to optimize task comprehensibility as well as monitoring and individualized adaptation of task demands. Alternative hardware and software solutions should be developed and/or investigated to provide more feasible options for implementing “Brain-IT” training. The “Brain-IT” training itself was well-accepted by older adults who have mNCD. Therefore, the investigation of the effectiveness of the “Brain-IT” training concept in a future RCT is warranted.

## Data availability statement

The datasets presented in this study can be found in online repositories. The names of the repository/repositories and accession number(s) can be found below: https://doi.org/10.5281/zenodo.7428378.

## Ethics statement

The studies involving humans were approved by ETH Zurich Ethics Committee ETH Zurich Office of Research Weinbergstrasse 11 WEC E 15/17 8092 Zürich. The studies were conducted in accordance with the local legislation and institutional requirements. The participants provided their written informed consent to participate in this study.

## Author contributions

PM was responsible for the conception of the study under the supervision of EB. PM was responsible for participant recruitment, data collection, statistical analysis, and writing the manuscript. HP contributed to EEG data collection and analysis. All authors contributed to the revisions of the manuscript, read, and approved the submitted version of the manuscript.
