# Supplementary material for: Feasibility, usability, and acceptance of “Brain-IT”—A newly developed exergame-based training concept for the secondary prevention of mild neurocognitive disorder: a pilot randomized controlled trial
Source: Front Aging Neurosci. 2023 Sep 21;15:1163388. doi: 10.3389/fnagi.2023.1163388 (PMC10557950; doi:10.3389/fnagi.2023.1163388)
Supplement: Supplementary file 1 [file Data_Sheet_1.PDF]

Supplementary Material

Feasibility, Usability and Acceptance of 'Brain-IT' - A Newly Developed Exergame-Based Training Concept for the Secondary Prevention of Mild Neurocognitive Disorder: A Pilot Randomized Controlled Trial

Patrick Manser*, Hanna Poikonen, Eling D. de Bruin

[1]Motor Control and Learning Group - Institute of Human Movement Sciences and Sport, ETH Zurich, Zurich, Switzerland;

[2]Learning Sciences and Higher Education, Department of Humanities, Social and Political Sciences, ETH Zurich, Switzerland;

[3]Department of Health, OST - Eastern Swiss University of Applied Sciences, St. Gallen, Switzerland;

[4]Division of Physiotherapy, Department of Neurobiology, Care Sciences and Society, Karolinska Institute, Stockholm, Sweden

16-digit ORCID and e-mail of the author(s):

Patrick Manser	ORCID: 0000-0003-3300-6524	patrick.manser@hest.ethz.ch
Hanna Poikonen	ORCID: 0000-0001-7337-7042	hanna.poikonen@gess.ethz.ch
Eling D. de Bruin	ORCID: 0000-0002-6542-7385	eling.debruin@hest.ethz.ch

*** Correspondence:**
Patrick Manser
ETH Zurich | Department of Health Sciences and Technology
Institute of Human Movement Sciences and Sport
Motor Control and Learning Group
HCP H24.3
Leopold-Ruzicka-Weg 4 | 8093 Zurich | Switzerland
Tel.: +41 79 519 96 46
E-mail: patrick.manser@hest.ethz.ch

1 Supplementary File 1 – CONSORT 2010 checklist

Table 1: CONSORT 2010 checklist of information to include when reporting a pilot or feasibility trial [1]

Section/Topic:	Item No:	Checklist item:	Reported in section(s):
TITLE AND ABSTRACT:			
	1a	Identification as a pilot or feasibility randomized trial in the title	'Title'
	1b	Structured summary of pilot trial design, methods, results, and conclusions (for specific guidance see CONSORT abstract extension for pilot trials)	'Abstract'
INTRODUCTION:			
Background and objectives:	2a	Scientific background and explanation of rationale for future definitive trial, and reasons for randomized pilot trial	'Introduction-Background' and 'Introduction-Prior Work'
	2b	Specific objectives or research questions for pilot trial	'Introduction-Objectives'
METHODS:			
Trial design:	3a	Description of pilot trial design (such as parallel, factorial) including allocation ratio	'Materials and Methods - Trial Design and Study Setting'
	3b	Important changes to methods after pilot trial commencement (such as eligibility criteria), with reasons	'Materials and Methods - Important Changes to the Trial Design and Study Setting after Commencement'
Participants:	4a	Eligibility criteria for participants	'Materials and Methods – Eligibility Criteria'
	4b	Settings and locations where the data were collected	'Materials and Methods – Outcomes' and Supplementary File 3
	4c	How participants were identified and consented	'Materials and Methods – Recruitment'
Interventions:	5	The interventions for each group with sufficient details to allow replication, including how and when they were actually administered	'Materials and Methods – Interventions'
Outcomes:	6a	Completely defined prespecified assessments or measurements to address each pilot trial objective specified in 2b, including how and when they were assessed	'Materials and Methods – Outcomes'
	6b	Any changes to pilot trial assessments or measurements after the pilot trial commenced, with reasons	N/A
	6c	If applicable, prespecified criteria used to judge whether, or how, to proceed with future definitive trial	'Materials and Methods – Outcomes'
Sample size:	7a	Rationale for numbers in the pilot trial	'Materials and Methods – Sample Size'
	7b	When applicable, explanation of any interim analyses and stopping guidelines	N/A (see Materials and Methods – Analytical Methods'
Randomization:			
Sequence generation:	8a	Method used to generate the random allocation sequence	'Materials and Methods – Randomization – Sequence Generation'
	8b	Type of randomisation(s); details of any restriction (such as blocking and block size)	'Materials and Methods – Randomization – Sequence Generation'
Allocation concealment mechanism:	9	Mechanism used to implement the random allocation sequence (such as sequentially numbered containers), describing any steps taken to conceal the sequence until interventions were assigned	'Materials and Methods – Randomization – Allocation Concealment Mechanism'

Implementation:	10	Who generated the random allocation sequence, who enrolled participants, and who assigned participants to interventions	'Materials and Methods – Randomization – Implementation
Blinding:	11a	If done, who was blinded after assignment to interventions (for example, participants, care providers, those assessing outcomes) and how	'Materials and Methods – Blinding'
	11b	If relevant, description of the similarity of interventions	N/A
Statistical methods:	12	Methods used to address each pilot trial objective whether qualitative or quantitative	'Materials and Methods – Statistical Methods'
RESULTS:			
Participant flow (a diagram is strongly recommended):	13a	For each group, the numbers of participants who were approached and/or assessed for eligibility, randomly assigned, received intended treatment, and were assessed for each objective	'Results – Recruitment and Participant Flow'
	13b	For each group, losses and exclusions after randomization, together with reasons	'Results – Recruitment and Participant Flow'
Recruitment:	14a	Dates defining the periods of recruitment and follow-up	'Results – Recruitment and Participant Flow'
	14b	Why the pilot trial ended or was stopped	'Results – Recruitment and Participant Flow'
Baseline data:	15	A table showing baseline demographic and clinical characteristics for each group	'Results – Baseline Data'
Numbers analyzed:	16	For each objective, number of participants (denominator) included in each analysis. If relevant, these numbers should be by randomized group	Primary Outcomes: 'Results – Primary Outcomes' and 'Figure 4 - Summary of the participant flow throughout the study' Secondary Outcomes: 'Results – Secondary Outcomes', 'Figure 4 - Summary of the participant flow throughout the study', and 'Table 8 - Statistics for all secondary outcomes'
Outcomes and estimation:	17	For each objective, results including expressions of uncertainty (such as 95% confidence interval) for any estimates. If relevant, these results should be by randomized group	Primary Outcomes: 'Results – Primary Outcomes' and 'Figure 4 - Summary of the participant flow throughout the study' Secondary Outcomes: 'Results – Secondary Outcomes', 'Figure 4 - Summary of the participant flow throughout the study', and 'Table 8 - Statistics for all secondary outcomes'
Ancillary analyses:	18	Results of any other analyses performed that could be used to inform the future definitive trial	N/A
Harms:	19	All important harms or unintended effects in each group (for specific guidance see CONSORT for harms)	'Results – Recruitment and Participant Flow'
	19a	If relevant, other important unintended consequences	N/A
DISCUSSION:			
Limitations:	20	Pilot trial limitations, addressing sources of potential bias and remaining uncertainty about feasibility	'Discussion – Limitations'
Generalizability:	21	Generalizability (applicability) of pilot trial methods and findings to future definitive trial and other studies	not reported
Interpretation:	22	Interpretation consistent with pilot trial objectives and findings, balancing potential benefits and harms, and considering other relevant evidence	'Discussion – Feasibility', 'Discussion – Usability', 'Discussion – Acceptance', and 'Conclusion'
	22a	Implications for progression from pilot to future definitive trial, including any proposed amendments	'Discussion – Feasibility', 'Discussion – Usability', 'Discussion – Acceptance', and 'Conclusion'
OTHER INFORMATION:			
Registration:	23	Registration number for pilot trial and name of trial registry	'Abstract' and 'Materials and Methods - Trial Design and Study Setting'
Protocol:	24	Where the pilot trial protocol can be accessed, if available	N/A

Funding:	25	Sources of funding and other support (such as supply of drugs), role of funders	‘Funding and Acknowledgement’
Ethics:	26	Ethical approval or approval by research review committee, confirmed with reference number	‘Materials and Methods - Trial Design and Study Setting’

2 Supplementary File 2 – Interview Guide

Table 2: Themes and guiding questions discussed during semi-structured interviews with study participants

Themes:	Guiding Questions:
Experiences and desired adaptations of the training concept	What do you remember most about the training? Why?<br><br>Would you like to continue the training? What adjustments would be necessary to make the training optimal for you?
Perceived Usefulness	Did you find the training useful? If so, what benefits did you feel? Would you recommend this training to your colleagues and acquaintances?<br><br>Do you feel any changes (e.g. mental and physical abilities, well-being) as a result of the training? If so, how exactly do these changes manifest themselves?

3 Supplementary File 3 – Details on the Specific Assessments and Measurement Conditions of all Secondary Outcomes

3.1 Global Cognition

The German version of the Quick Mild Cognitive Impairment Screen (Qmci) was used to assess the global level of cognitive functioning [2, 3]. The Qmci has been validated against the standardized AD Assessment Scale-Cognitive Subscale (ADAS-Cog) [2, 4], which is considered the gold standard for assessing the efficacy of antidementia treatments [5-7]. Furthermore, it is accurate at differentiating mNCD from normal cognition and MNCD [4]. With this regard, is has also been externally validated and has shown a higher level of accuracy, sensitivity, and specificity than commonly used tests such as the (Standardised) Mini Mental State Exam ((S)MMSE) and the Montreal Cognitive Assessment (MoCA) at detecting cognitive impairment (MCI and dementia). In comparison to the (S)MMSE and MoCA, the Qmci includes a more detailed scoring system and a logical memory task that allow the Qmci to detect more subtle cognitive abnormalities and avoid ceiling effects. [8] The Qmci is scored as a point rate out of a maximum score of 100. It comprises six subtests: orientation (10 points), registration (5 points), clock drawing (15 points), delayed recall (20 points), verbal fluency (20 points), and logical memory (30 points) [3, 9]. The Qmci was administered and evaluated according to published guidelines [3].

3.2 Domain-Specific Cognitive Functioning

3.2.1 Learning and Memory

Learning and memory was assessed using the German version of the subtests 'logical memory' of the Wechsler Memory Scale-Revised (WMS-IV-LM) [10, 11] and a computerized version of the Digit Span Forward test (Psychology experiment building language (PEBL)-Digit Span Forward (PEBL-DSF)) [12-14].

The WMS-IV-LM measures auditory verbal contextual learning and memory with excellent reliability and validity [11]. The validated Older Adults battery (for ages 65 or older) of the German version of the WMS-IV-LM [10, 11, 15] was used for all participants. The test was instructed, conducted and evaluated according to the standardized administration and scoring manual [10]. During the 20 - 30 minutes retention phase, unrelated assessment (e.g. gait analysis, questionnaires) were performed that do not interfere with memory.

The PEBL-DSF test assesses immediate recall and was executed using the PEBL Test battery software (version 2.1 (2); with default settings) [12-14]. Participants had to remember and repeat digit sequences presented on the screen. Span length covered two to a maximum of eight digits. For each digit span, two trials were presented prior to increasing sequence length (in case at least one of the two trials was completed correctly). For every correct replication of a digit sequence, one point was scored, summing up to a total point score. Additionally, the length of the longest correctly repeated digit sequence was recorded as the maximum span. Instructions were presented on the screen and were explained verbally to each participant before starting the test.

3.2.2 Complex Attention

Complex attention was assessed using a computerized version of the Trail Making Test – Part A (PEBL-TMT-A) [12] and the subtests ‘Alertness’, ‘Go-NoGo’ of the Test of Attentional Performance (TAP Alertness, TAP Go-NoGo) [16].

The TMT-A is valid and reliable neuropsychological tests to assess psychomotor processing speed and visuo-perceptual abilities [17-22]. A computerized version of the of TMT-A (PEBL Test battery software (version 2.1 (2); with default settings) was used [12, 14, 23]. Participants were instructed verbally, and a short practice session was conducted before starting the test. Completion time was limited to 300 seconds. Completion times [s] (including the time for correction of errors) and number of errors were measured.

The test of attentional performance (TAP; TAP version 2.3.1, PSYTEST, Psychologische Testsysteme, Herzogenrath, Germany) is a valid and reliable computerized test battery to assess various attentional and executive functions [16, 24], with norm values for healthy older adults provided by the supplier [16, 25]. The TAP Alertness was designed to determine intrinsic and phasic attention under different conditions. The first condition (A) required participants to react to a cross appearing at randomly varying intervals on the screen by pressing a switch as fast as possible. In the second task (B), responses were only required when preceded by a warning tone. Twenty target stimuli were presented for each condition in the order ‘ABBA’ to compensate for the effects of fatigue. Median reaction times were measured. [16] The TAP Go-NoGo was used to assess selective attention and inhibition. The test form ‘1 of 2’ was instructed, conducted and evaluated according to the standardized protocol of the manufacturer. Median reaction times and number of errors were measured. [16]

3.2.3 Executive Function

Executive Function was assessed considering planning (i.e. using the HOTAP picture-sorting test part A (HOTAP-A) [26]), working memory (i.e. using a computerized version of the Digit Span Backward test (PEBL Digit Span Backward (PEBL-DSB)) [12-14], inhibition (subtests ‘Incompatibility’ of the Test of Attentional Performance (TAP Incompatibility) [16]), and cognitive flexibility (i.e. using a computerized version of the Trail Making Test – Part B (PEBL-TMT-B) [12, 14]).

To measure planning ability, the HOTAP-A [26] was used. A set of photo cards containing actions typical for everyday life (e.g. making coffee, washing clothes, grocery shopping) were presented. The participants were verbally instructed to sort photo cards on which individual sub-steps of these typical everyday actions are depicted. The test was instructed, conducted, and evaluated according to the standardized protocol of the manufacturer. The outcome variable was a combi score calculated by the sum of points divided by the time needed to arrange the cards. [26]

The PEBL Digit Span Backward (PEBL-DSB) [12-14] test was used to assess short-term working memory capacity. It was instructed, administered, and scored identical to the PEBL-DSF, but participants had to remember and repeat digit sequences in reverse order.

The subtest TAP Incompatibility was used to assess cognitive inhibition. The test was instructed, conducted and evaluated according to the standardized protocol of the manufacturer. Median reaction times and the number of mistakes were measured for each condition (i.e. compatible and incompatible) [16].

The TMT-B is valid and reliable neuropsychological tests to assess cognitive flexibility [17-22]. It consists of 25 randomly allocated circles distributed over a sheet of paper. A computerized version of the of the TMT-A (PEBL Test battery software (version 2.1 (2); with default settings) was used in this study [12, 14, 23]. It was instructed, administered, and scored identical to the PEBL-TMT-A.

3.2.4 Visuospatial skills

Visuo-spatial skills were tested with a computerized version of the classic Shepard and Metzler's mental rotation task [27]. The PEBL-Mental Rotation Task (PEBL-MRT) was executed using the PEBL Test battery software (version 2.1 (2); with default settings) [12, 14, 28]. Instructions were presented on the screen and were explained verbally to each participant before starting the task. Pairs of differently rotated two-dimensional polygons were presented simultaneously on the screen. Participants needed to decide as quickly as possible whether the two presented objects are identical (i.e. pressing <Lshift> on the keyboard) or different (i.e. pressing <Rshift> on the keyboard). Median reaction time of correct answered trials [s] as well as performance (number of correct answered trials) were assessed as an indicator for mental rotation ability [27, 28]. Trials with reaction times of less than 0.2 seconds or greater than 13 seconds were excluded from data analysis [28].

3.3 Resting-state Cortical Activity measured with Electroencephalography (EEG)

3.3.1 Data Acquisition:

Resting-state EEG activity was recorded at a sampling frequency of 1,000 Hz by a high-density 64-channel EEG system (eego sport, ANT Neuro, Enschede, The Netherlands). Three EEG cap sizes were employed in order to accommodate different head circumferences (waveguard, ANT Neuro, Enschede, The Netherlands) The electrode placement scheme by ANT Neuro (an extension to the 10/20 and 10/10 systems) was used [29]. All electrodes were referenced to Fz, whereas Pz was used as ground electrode. We aimed to reach electrodes impedances ≤ 10 kΩ for most electrodes before starting EEG recordings. The measurement was conducted at the same conditions as the measurement of resting heart rate and HRV. The measurement took place in the resting awake state; two repeats of two minutes eyes closed, two minutes eyes opened, resulting in a total measurement duration of eight minutes. Only the results related to the eyes closed condition are reported which is in line with previous research [30, 31].

3.3.2 Data Pre-Processing:

EEG data analysis was performed using custom scripts written in MATLAB R2021b (The Mathworks, Natick, MA, United States) and using the EEGLAB v2021.1 open-source toolbox [32]. First, EEG raw data was re-referenced using the average of all the EEG electrodes as a reference. Second, the data was high- and low-pass filtered (Finite impulse response (FIR) filtering, based on the firls (least square fitting of FIR coefficients) with a cut-off of the frequency pass band (Hz) of 0.5 Hz and 40 Hz, respectively) using the function pop_eegfilt. Third, we checked for bad channels. Invalid EEG channels with more than 5 seconds of flat line signal or having a correlation less than 0.4 with surrounding channel locations were excluded using the function clean_rawdata. Fourth, we ran an Independent Component Analysis (ICA) decomposition using the 'runica' algorithm to detect and remove artefacts related to eye movements and blinks visually inspecting and labeling the individual ICA components by map. Fifth, bad channels were interpolated using the function eeg_interp and the method 'invdist' (inverse distance on the scalp). Sixth, the data was epoched. We separated the data in four epochs of 100 seconds, each representing the middle 100 seconds of each of the four 120 seconds measurement conditions (i.e. two repeats of two minutes eyes closed, two minutes eyes opened).

3.3.3 Spectral Analysis:

In patients with cognitive impairment (i.e. mild to major NCD; especially in Alzheimer's disease patients), a slowdown of EEG signals indicated by a power spectrum shifts from high-frequency components (alpha, beta, and gamma) towards low- frequency components (delta and theta) is commonly observed [33-37]. An increase in theta frequency is one of the important biomarkers in both mild to major NCD [30]. Poil and colleagues (2013) reported local changes in beta power over the electrode Cz in mild cognitive impairment in comparison to healthy controls [31]. The area(s) of slow wave activity were shown to be primarily localized to the (left) temporal regions [30, 38] and have been shown to be consistent with neuropsychological assessment in patients with NCD [38]. Additionally, the slowing of the EEG seems to be related to the extent of brain damage and proportional to the disease progression [34, 38]. In particular, correlations between the progressive atrophy of the hippocampus and the decrease in the cortical alpha frequency power, as well as the frontal white matter and the amplitude of delta frequencies in the frontal area have been reported [36]. Based on these findings, a spectral analysis of the cleaned and preprocessed data was computed using Welch's method to transform the data of all channels into the theta (4 – 8 Hz) and beta (13 – 30 Hz) frequency bands and have selected the following areas of interest for further analysis: beta band: central (Cz) [31] and theta band: left temporal (T7), right temporal (T8), left frontotemporal (FT7), and right frontotemporal (FT8) [30].

3.3.4 Analysis of Phase-Synchrony:

In addition to a slowdown of EEG signals, a decrease in synchronization, manifested as a reduction in connectivity between cortical regions, is often observed in patients with cognitive impairment (i.e. mild to major NCD; especially in Alzheimer's disease patients) [33, 34, 36, 37]. The cause behind the reduced connectivity is not yet well understood, although it may be attributed to cortical atrophy in the communication of neural networks [34, 39, 40] and functional disconnection of the neocortex [37]. Therefore, we calculated the phase synchrony index γ [41] based on the Hilbert transform of the alpha (8 – 13 Hz) band-pass filtered EEG signal [42]. Phase synchrony analysis was conducted on alpha frequency over the fronto-temporal electrode pairs Fp2-C4, F7-T6, T3-T6 and T5-T6 which were altered in mild cognitive impairment in comparison to healthy controls in a previous study [30].

3.4 Spatiotemporal Parameters of Gait

Spatiotemporal gait parameters were assessed using a BTS G-WALK® (BTS Bioengineering S.p.A., Garbagnate Milanese, Italy) inertial sensor attached with semi-elastic belt to the lower back of the participant. The BTS G-WALK® sensor delivers valid [43-45] and reliable [43, 46] spatiotemporal gait parameters. All acceleration data were sampled at a frequency of 100 Hz. Data was transmitted for analysis through a Bluetooth® 3.0 connection to the software program BTS G-Studio (BTS Bioengineering S.p.A., Italy). A gait-analysis protocol consisting of a figure of eight walking path (i.e. distance between cones approximately 8 m) was applied [47]. At least 50 consecutive gait cycles are needed to ensure reliability of spatial and temporal parameters of gait variability [48]. Therefore, participants did – depending on their walking speed and stride length - perform five to ten repetitions of the figure of eight walking path at preferred walking speed. Comparative quantitative reference values for healthy older adults are available [49]. Gait speed at preferred walking speed [$m \cdot s^{-1}$], stride duration [ms], stride length [cm], stance phase duration [% stride duration], swing phase duration [% stride duration], single support time [%], and double support time [%] were evaluated as outcome variables.

3.5 Psychosocial Factors

Quality of Life (QoL) was evaluated in interview format using the Quality of Life-Alzheimer's Disease (QOL-AD) scale [50]. The QOL-AD is a valid and reliable self-report 13-item scale assessing various domains of QOL of cognitively impaired patients [50, 51]. The German version of the QOL-AD scale, that has a high test-retest reliability and good construct validity [52, 53], was used. Administration and evaluation followed standardized instructions [53, 54]. Comparable values for individuals with mNCD are available [55].

Levels of depression, anxiety, and stress was assessed using the short version of the Depression, Anxiety and Stress Scale-21 (DASS-21) [56-58]. DASS-21 has a high reliability, good convergent and discriminant validity [56, 59]. The validated German version of the DASS-21 was administered and scored according the guidelines and scoring template [60, 61]. Normative data of the three subscales are available and suggest cut-off scores of 10, 8, or 15 indicating significant depression, anxiety, or stress, respectively [60]. Comparative values for individuals with mNCD are available [62].

3.6 Cardiac Vagal Modulation (Resting vagally-mediated Heart Rate Variability (vm-HRV))

To determine resting vm-HRV, all participants were instructed to sit in a comfortable position on a chair without speaking, both feet flat on the floor with knees at a 90° angle, hands on thighs (i.e. palms facing upward), and eyes closed [63]. The measurement was performed in a quiet room with dimmed light and at room temperature with a heart rate monitor (Polar M430) and sensor (Polar H10). The initial acclimatization phase lasted for 5 min followed by a 5 min resting measurement, the recommended standard duration for short-term recordings [63, 64]. The start of the recording was not announced to participants [63]. Data was collected with a sampling rate of 1000 Hz to provide a temporal resolution of 1 ms for each R–R interval [65]. R-R data recordings were directly transmitted to Kubios HRV Premium (Kubios Oy, Kuopio, Finland, version 3.4) for analysis. Kubios HRV is a scientifically validated software for HRV analysis and has achieved a gold-standard status in research [66-69]. The automatic beat correction algorithm and noise handling provided by the software was used to correct for artifact and/or ectopic beats. The algorithm was validated for measurements at rest [66]. After removing inter-beat-interval time series non-stationarities by detrending analysis using the smoothness priors method approach (settings: detrending method = smoothn priors, Lambda = 500, f_c = 0.035 Hz), mean values of mainly vagal-mediated HRV indices were calculated for each segment. For that purpose, the mean R-R time interval (mRR) [ms], root mean square of successive RR interval differences (RMSSD) [ms], the percentage of successive RR intervals that differ by more than 50 ms (pNN50) [%], the absolute power of the high-frequency (0.15 – 0.4 Hz; HF) band [ms^2], the relative power of HF (in normal units; HF [n.u.] = HF [ms^2] / (total power [ms^2] – very low frequency (0.00 – 0.04 Hz [ms^2])), and the Poincaré plot standard deviation perpendicular to the line of identity (SD1) [ms] were considered [63, 64, 70-72]. Additionally, the parasympathetic nervous system tone index (PNS-Index) [] was calculated that compares parasympathetic nervous system (PNS) activity to normal resting values [72].

4 References

1. Eldridge, S.M., et al., CONSORT 2010 statement: extension to randomised pilot and feasibility trials. Bmj-British Medical Journal, 2016. 355.
2. O'Caoimh, R., The Quick Mild Cognitive Impairment (Qmci) screen: developing a new screening test for mild cognitive impairment and dementia. 2015, University College Cork.
3. O'Caoimh, R. and D.W. Molloy, The Quick Mild Cognitive Impairment Screen (Qmci), in Cognitive Screening Instruments. 2017. p. 255-272.
4. O'Caoimh, R., et al., *The Quick Mild Cognitive Impairment screen correlated with the Standardized Alzheimer's Disease Assessment Scale–cognitive section in clinical trials.* Journal of Clinical Epidemiology, 2014. **67**(1): p. 87-92.
5. Kueper, J.K., M. Speechley, and M. Montero-Odasso, *The Alzheimer's Disease Assessment Scale-Cognitive Subscale (ADAS-Cog): Modifications and Responsiveness in Pre-Dementia Populations. A Narrative Review.* J Alzheimers Dis, 2018. **63**(2): p. 423-444.
6. Rosen, W.G., R.C. Mohs, and K.L. Davis, *A new rating scale for Alzheimer's disease.* American Journal of Psychiatry, 1984. **141**(11): p. 1356-1364.
7. Mohs, R.C., et al., *Development of cognitive instruments for use in clinical trials of antidementia drugs: additions to the Alzheimer's Disease Assessment Scale that broaden its scope. The Alzheimer's Disease Cooperative Study.* Alzheimer Dis Assoc Disord, 1997. **11 Suppl 2**: p. S13-21.
8. Glynn, K., R. Coen, and B.A. Lawlor, *Is the Quick Mild Cognitive Impairment Screen (QMCI) more accurate at detecting mild cognitive impairment than existing short cognitive screening tests? A systematic review of the current literature.* Int J Geriatr Psychiatry, 2019. **34**(12): p. 1739-1746.
9. O'Caoimh, R., et al., *Comparison of the quick mild cognitive impairment (Qmci) screen and the SMMSE in screening for mild cognitive impairment.* Age and Ageing, 2012. **41**(5): p. 624-629.
10. Petermann, F. and A.C. Lepach, *Wechsler Memory Scale® – Fourth Edition (WMS®-IV) - Manual zur Durchführung und Auswertung (Deutsche Übersetzung und Adaptation der WMS®-IV von David Wechsler)*. 2012: Pearson Assessment and Information GmbH.
11. Wechsler, D., *Wechsler memory scale–fourth edition (WMS-IV).* New York, NY: The Psychological Corporation, 2009.
12. Mueller, S.T. and B.J. Piper, *The Psychology Experiment Building Language (PEBL) and PEBL Test Battery.* Journal of Neuroscience Methods, 2014. **222**: p. 250-259.
13. Croschere, J., et al., *The effects of time of day and practice on cognitive abilities: Forward and backward Corsi block test and digit span.* PEBL Technical Report Series, 2012.
14. Mueller, S.T. *PEBL: The Psychology experiment building language (Version 0.14) [Computer experiment programming language]*. 2014 [cited 2020 January]; Available from: http://pebl.sourceforge.net.
15. Lepach, A.C. and F. Petermann, *Gedächtnisdiagnostik mit der Wechsler Memory Scale – Fourth Edition.* Zeitschrift für Neuropsychologie, 2012. **23**(3): p. 123-132.

16. Zimmermann, P. and B. Fimm, *Testbatterie zur Aufmerksamkeitsprüfung-Version 2.3 [A test battery for attentional performance-Version 2.3]*. Herzogenrath: Psychologische Testsysteme, 2012.

17. Bowie, C.R. and P.D. Harvey, *Administration and interpretation of the Trail Making Test.* Nat Protoc, 2006. **1**(5): p. 2277-81.

18. Tombaugh, T.N., *Trail Making Test A and B: normative data stratified by age and education.* Arch Clin Neuropsychol, 2004. **19**(2): p. 203-14.

19. Reitan, R.M., *Validity of the Trail Making Test as an Indicator of Organic Brain Damage.* Perceptual and Motor Skills, 2016. **8**(3): p. 271-276.

20. Gaudino, E.A., M.W. Geisler, and N.K. Squires, *Construct validity in the Trail Making Test: what makes Part B harder?* J Clin Exp Neuropsychol, 1995. **17**(4): p. 529-35.

21. Sanchez-Cubillo, I., et al., *Construct validity of the Trail Making Test: role of task-switching, working memory, inhibition/interference control, and visuomotor abilities.* J Int Neuropsychol Soc, 2009. **15**(3): p. 438-50.

22. Reitan, R. and D. Wolfson, *The Halstead-Reitan Cognitive Test Battery: Theory and Clinical Interpretation*. 1993, Neuropsychology Press, Tucson, AZ.

23. Piper, B.J., et al., *Executive function on the Psychology Experiment Building Language tests.* Behav Res Methods, 2012. **44**(1): p. 110-23.

24. Zimmermann, P. and B. Fimm, *A test battery for attentional performance*, in *Applied neuropsychology of attention*. 2004, Psychology Press. p. 124-165.

25. Zimmermann, P.F., B. *Norms - TAP 2.3.1 - Norms of tests and subtests*. 2017. **2019**.

26. Menzel-Begemann, A., *HOTAP–Handlungsorganisation und Tagesplanung.* Testverfahren zur Erfassung der Planungsfähigkeit im Alltag: Göttingen, 2009.

27. Shepard, R.N. and J.J.S. Metzler, *Mental rotation of three-dimensional objects.* 1971. **171**(3972): p. 701-703.

28. Berteau-Pavy, D., J. Raber, and B. Piper, *Contributions of age, but not sex, to mental rotation performance in a community sample*. 2011, sn]. Disponível em:< http://sites. google. com/site/pebltechnicalreports ….

29. Chatrian, G.E., E. Lettich, and P.L. Nelson, *Ten Percent Electrode System for Topographic Studies of Spontaneous and Evoked EEG Activities.* American Journal of EEG Technology, 2015. **25**(2): p. 83-92.

30. Meghdadi, A.H., et al., *Resting state EEG biomarkers of cognitive decline associated with Alzheimer's disease and mild cognitive impairment.* PLoS One, 2021. **16**(2): p. e0244180.

31. Poil, S.-S., et al., *Integrative EEG biomarkers predict progression to Alzheimer's disease at the MCI stage.* Frontiers in Aging Neuroscience, 2013. **5**.

32. Delorme, A. and S. Makeig, *EEGLAB: an open source toolbox for analysis of single-trial EEG dynamics including independent component analysis.* Journal of Neuroscience Methods, 2004. **134**(1): p. 9-21.

33. Al-Qazzaz, N.K., et al., *Role of EEG as Biomarker in the Early Detection and Classification of Dementia.* Scientific World Journal, 2014. **2014**: p. 906038.

34. Cassani, R., et al., *Systematic Review on Resting-State EEG for Alzheimer's Disease Diagnosis and Progression Assessment.* Disease Markers, 2018. **2018**: p. 5174815.

35. Micanovic, C. and S. Pal, *The diagnostic utility of EEG in early-onset dementia: a systematic review of the literature with narrative analysis.* J Neural Transm (Vienna), 2014. **121**(1): p. 59-69.

36. Dauwels, J., F. Vialatte, and A. Cichocki, *Diagnosis of Alzheimers Disease from EEG Signals: Where Are We Standing?* Current Alzheimer Research, 2010. **7**(6): p. 487-505.

37. Dauwels, J., F.-B. Vialatte, and A. Cichocki. *On the Early Diagnosis of Alzheimer's Disease from EEG Signals: A Mini-Review*. 2011. Dordrecht: Springer Netherlands.

38. Malek, N., et al., *Electroencephalographic markers in dementia.* Acta Neurol Scand, 2017. **135**(4): p. 388-393.

39. Wen, D., Y. Zhou, and X. Li, *A critical review: coupling and synchronization analysis methods of EEG signal with mild cognitive impairment.* Front Aging Neurosci, 2015. **7**: p. 54.

40. Babiloni, C., et al., *Brain neural synchronization and functional coupling in Alzheimer's disease as revealed by resting state EEG rhythms.* International Journal of Psychophysiology, 2016. **103**: p. 88-102.

41. Lachaux, J.P., et al., *Measuring phase synchrony in brain signals.* Hum Brain Mapp, 1999. **8**(4): p. 194-208.

42. Tass, P., et al., *Detection of n:m Phase Locking from Noisy Data: Application to Magnetoencephalography.* Physical Review Letters, 1998. **81**(15): p. 3291-3294.

43. Ridder, R.D., et al., *Concurrent Validity of a Commercial Wireless Trunk Triaxial Accelerometer System for Gait Analysis.* Journal of Sport Rehabilitation, 2019. **28**(6).

44. Park, G. and Y. Woo, *Comparison between a center of mass and a foot pressure sensor system for measuring gait parameters in healthy adults.* Journal of Physical Therapy Science, 2015. **27**(10): p. 3199-3202.

45. Vítečková, S., et al., *Agreement between the GAITRite® System and the Wearable Sensor BTS G-Walk® for measurement of gait parameters in healthy adults and Parkinson's disease patients.* PeerJ, 2020. **8**: p. e8835.

46. YAZICI, G., et al., *The reliability of a wearable movement analysis system (G-walk) on gait and jump assessment in healthy adults.* Journal of Exercise Therapy and Rehabilitation, 2020. **7**(2): p. 159-167.

47. Bioengineering, B. *G-WALK User manual english version 8.1.0*. 2017.

48. Konig, N., et al., *Is gait variability reliable? An assessment of spatio-temporal parameters of gait variability during continuous overground walking.* Gait Posture, 2014. **39**(1): p. 615-7.

49. Beauchet, O., et al., *Guidelines for Assessment of Gait and Reference Values for Spatiotemporal Gait Parameters in Older Adults: The Biomathics and Canadian Gait Consortiums Initiative.* Front Hum Neurosci, 2017. **11**(353): p. 353.

50. Gibbons, L., S. McCurry, and L. Teri, *Quality of Life in Alzheimer's disease: Patient and Caregiver Reports.* Journal of Mental Health and Aging, 1999. **5**: p. 21-32.

51. Logsdon, R.G., et al., *Assessing quality of life in older adults with cognitive impairment.* Psychosom Med, 2002. **64**(3): p. 510-9.

52. Stypa, V., et al., *Validity and Reliability of the German Quality of Life-Alzheimer's Disease (QoL-AD) Self-Report Scale.* J Alzheimers Dis, 2020. **77**(2): p. 581-590.

53. Trust, M.R. *Lebensqualität von Personen mit Alzheimer-Krankheit (QOL-AD)*. 2016.

54. Trust, M.R. *Quality Of Life in Alzheimer's Disease Version 1.1 Scaling and Scoring*. 2019.

55. Bárrios, H., et al., *Quality of life in patients with mild cognitive impairment.* Aging & Mental Health, 2013. **17**(3): p. 287-292.

56. Henry, J.D. and J.R. Crawford, *The short-form version of the Depression Anxiety Stress Scales (DASS-21): Construct validity and normative data in a large non-clinical sample.* British Journal of Clinical Psychology, 2005. **44**(Pt 2): p. 227-239.

57. Lovibond, S.H. and P.F. Lovibond, *Manual for the depression anxiety stress scales*. 1996: Psychology Foundation of Australia.

58. Lovibond, P.F. and S.H. Lovibond, *The Structure of Negative Emotional States - Comparison of the Depression Anxiety Stress Scales (Dass) with the Beck Depression and Anxiety Inventories.* Behaviour Research and Therapy, 1995. **33**(3): p. 335-343.

59. Gloster, A.T., et al., *Psychometric properties of the Depression Anxiety and Stress Scale-21 in older primary care patients.* J Affect Disord, 2008. **110**(3): p. 248-59.

60. Nilges, P. and C. Essau, *Die Depressions-Angst-Stress-Skalen.* Der Schmerz, 2015. **29**(6): p. 649-657.

61. Nilges, P. and C. Essau, *DASS. Depressions-Angst-Stress-Skalen-deutschsprachige Kurzfassung.* 2021.

62. Gates, N., et al., *Psychological well-being in individuals with mild cognitive impairment.* Clin Interv Aging, 2014. **9**: p. 779-92.

63. Laborde, S., E. Mosley, and J.F. Thayer, *Heart Rate Variability and Cardiac Vagal Tone in Psychophysiological Research - Recommendations for Experiment Planning, Data Analysis, and Data Reporting.* Front Psychol, 2017. **8**: p. 213.

64. Malik, M., *Heart rate variability: Standards of measurement, physiological interpretation, and clinical use.* Circulation, 1996. **93**: p. 1043-1065.

65. Williams, D.P., et al., *Two-week test-retest reliability of the Polar® RS800CX™ to record heart rate variability.* Clinical Physiology and Functional Imaging, 2017. **37**(6): p. 776-781.

66. Lipponen, J.A. and M.P. Tarvainen, *A robust algorithm for heart rate variability time series artefact correction using novel beat classification.* J Med Eng Technol, 2019. **43**(3): p. 173-181.

67. Niskanen, J.P., et al., *Software for advanced HRV analysis.* Computer Methods and Programs in Biomedicine, 2004. **76**(1): p. 73-81.

68. Tarvainen, M.P., et al., *Kubios HRV - Heart rate variability analysis software.* Computer Methods and Programs in Biomedicine, 2014. **113**(1): p. 210-220.

69. Tarvainen, M.P., P.O. Ranta-Aho, and P.A. Karjalainen, *An advanced detrending method with application to HRV analysis.* IEEE Trans Biomed Eng, 2002. **49**(2): p. 172-5.

70. Shaffer, F. and J.P. Ginsberg, *An Overview of Heart Rate variability Metrics and Norms.* Frontiers in Public Health, 2017. **5**: p. 258-258.

71. Ernst, G., *Heart-Rate Variability-More than Heart Beats?* Front Public Health, 2017. **5**: p. 240.

72. Mika P. Tarvainen, P.D.J.L., PhD Juha-Pekka Niskanen, PhLic Perttu O. Ranta-aho, MSc *Kubios HRV (ver. 3.4) USER'S GUIDE*. 2018.
